# Identification and validation of senescence-related genes in circulating endothelial cells of patients with acute myocardial infarction

**DOI:** 10.3389/fcvm.2022.1057985

**Published:** 2022-12-13

**Authors:** Jie Xiang, Jun Shen, Ling Zhang, Baopeng Tang

**Affiliations:** ^1^Xinjiang Key Laboratory of Cardiac Electrophysiology and Remodeling, The First Affiliated Hospital of Xinjiang Medical University, Ürümqi, China; ^2^Department of Pacing and Electrophysiology, The First Affiliated Hospital of Xinjiang Medical University, Ürümqi, China

**Keywords:** acute myocardial infarction, cellular senescence-related genes, biological markers, bioinformatics, aging

## Abstract

**Background:**

Acute myocardial infarction (AMI) is the main clinical cause of death and cardiovascular disease and thus has high rates of morbidity and mortality. The increase in cardiovascular disease with aging is partly the result of vascular endothelial cell senescence and associated vascular dysfunction. This study was performed to identify potential key cellular senescence-related genes (SRGs) as biomarkers for the diagnosis of AMI using bioinformatics.

**Methods:**

Using the CellAge database, we identified cellular SRGs. GSE66360 and GSE48060 for AMI patients and healthy controls and GSE19322 for mice were downloaded from the Gene Expression Omnibus (GEO) database. The GSE66360 dataset was divided into a training set and a validation set. The GSE48060 dataset was used as another validation set. The GSE19322 dataset was used to explore the evolution of the screened diagnostic markers in the dynamic process of AMI. Differentially expressed genes (DEGs) of AMI were identified from the GSE66360 training set. Differentially expressed senescence-related genes (DESRGs) selected from SRGs and DEGs were analyzed using Gene Ontology (GO) enrichment, Kyoto Encyclopedia of Genes and Genomes (KEGG) pathways, and protein-protein interaction (PPI) networks. Hub genes in DESRGs were selected based on degree, and diagnostic genes were further screened by gene expression and receiver operating characteristic (ROC) curve. Finally, a miRNA-gene network of diagnostic genes was constructed and targeted drug prediction was performed.

**Results:**

A total of 520 DEGs were screened from the GSE66360 training set, and 279 SRGs were identified from the CellAge database. The overlapping DEGs and SRGs constituted 14 DESRGs, including 4 senescence suppressor genes and 10 senescence inducible genes. The top 10 hub genes, including FOS, MMP9, CEBPB, CDKN1A, CXCL1, ETS2, BCL6, SGK1, ZFP36, and IGFBP3, were screened. Furthermore, three diagnostic genes were identified: MMP9, ETS2, and BCL6. The ROC analysis showed that the respective area under the curves (AUCs) of MMP9, ETS2, and BCL6 were 0.786, 0.848, and 0.852 in the GSE66360 validation set and 0.708, 0.791, and 0.727 in the GSE48060 dataset. In the GSE19322 dataset, MMP9 (AUC, 0.888) and ETS2 (AUC, 0.929) had very high diagnostic values in the early stage of AMI. Finally, based on these three diagnostic genes, we found that drugs such as acetylcysteine and genistein may be targeted for the treatment of age-related AMI.

**Conclusion:**

The results of this study suggest that cellular SRGs might play an important role in AMI. MMP9, ETS2, and BCL6 have potential as specific biomarkers for the early diagnosis of AMI.

## 1 Introduction

Acute myocardial infarction (AMI), a life-threatening disease caused by coronary atherosclerosis, is a leading cause of morbidity and mortality worldwide and places a huge burden on the global economy and health (1, 2). Early, rapid and accurate diagnosis of AMI is vital to initiate effective evidence-based medical management and treatment. Currently, the diagnosis of AMI mainly includes clinical evaluation, electrocardiography and cardiac troponin (cTn) testing (3). Percutaneous coronary intervention, antithrombotic therapy, and secondary prevention are currently the three major approaches in the clinical treatment of AMI (4). Despite significant advances in the prevention and treatment of AMI in recent decades, the incidence of myocardial infarction has not declined but has increased dramatically in an increasingly aged population (5, 6). When AMI occurs, early, timely and effective revascularization can reduce mortality and improve prognosis. However, the data indicate that approximately one-third of patients eligible for early reperfusion therapy are not diagnosed in time, thus missing the optimal treatment opportunity and leading to serious adverse events (7). Therefore, early and accurate diagnosis is particularly important to reduce the incidence of adverse events and improve overall survival, particularly in elderly individuals with atypical symptoms. Although many cardiovascular disease studies have considered both young and old individuals, there are still many unanswered questions regarding the interaction between age and AMI.

Aging is closely correlated with cardiovascular diseases such as atherosclerosis and thrombosis (8, 9). According to statistics, cardiovascular disease is the leading cause of death in the elderly population, accounting for approximately 25% of all deaths in the United States, and 81% of cardiovascular deaths occur in individuals over 65 years old (10). The pathological mechanism of aging that leads to atherosclerosis mainly includes endothelial damage and oxidative stress (8). Cellular senescence plays a crucial role in age-related organ dysfunction and can be induced by aging in a variety of cells, including endothelial cells, which produce a large number of vasosecretory factors that are essential for maintaining tissue homeostasis (11). Endothelial senescence increases with systemic aging and leads to cellular dysfunction and ultimately to cardiovascular diseases such as atherosclerosis (12), which can cause fatal AMI. In recent decades, the process of cellular senescence has been widely studied, and great progress has been made. A deeper study of endothelial senescence-related regulatory genes and mechanisms will further contribute to our understanding of the physiological significance and potential therapeutic applications of cellular senescence. However, the pathophysiology between endothelial senescence and AMI remains unclear.

In the present study, two datasets containing circulating endothelial cell and circulating cell gene expression profiles of AMI patients were downloaded from the Gene Expression Omnibus (GEO) database^1^ and divided into a training set and validation set according to age. In addition, cellular senescence-related genes (SRGs) were screened from the CellAge database.^2^ Next, differentially expressed genes (DEGs) and gene overlap between DEGs and SRGs was obtained. Next, differentially expressed senescence-related genes (DESRGs) were analyzed using bioinformatics methods, including Gene Ontology (GO) term enrichment, the Kyoto Encyclopedia of Genes and Genomes (KEGG), and protein-protein interaction (PPI) network analysis. We then used receiver operating characteristic (ROC) curves to evaluate the area under the curve (AUC) value and predictive ability of the hub genes to identify the diagnostic genes. Finally, diagnostic genes were used to predict potential therapeutic drugs.

## 2 Materials and methods

### 2.1 Data acquisition

**FIGURE 1:**
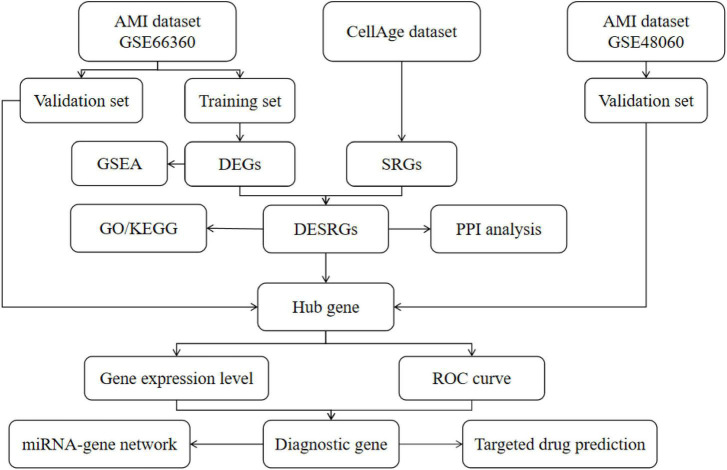
The flow chart of this study. DEGs, differentially expressed genes; SRGs, senescence-related genes; GSEA, gene set enrichment analysis; DESRGs, differentially expressed senescence-related genes; GO, Gene Ontology; KEGG, Kyoto Encyclopedia of Genes and Genomes; ROC, receiver operating characteristic.

A total of 279 SRGs were obtained from the CellAge database; all SRGs are listed in Supplementary Table 1. Microarray data, including GSE66360, GSE48060, and GSE19322, were downloaded from the NCBI GEO database. GPL570 [(HG-U133_Plus_2) Affymetrix Human Genome U133 Plus 2.0 Array] serves as the complementary microarray detection platform for both the GSE66360 and GSE48060 databases; GSE19322 was based on platform GPL339 [(MOE430A) Affymetrix Mouse Expression 430A Array]. The GSE66360 dataset, which describes the differences in circulating endothelial cells, was divided into a training set (21 AMI patients, mean age 59 years vs. 22 healthy controls, mean age 28.6 years) and a validation set (28 AMI patients vs. 28 healthy controls, mean age unknown). The GSE48060 dataset, which describes the differences in peripheral blood, was used as another validation set (31 AMI patients, mean age 56 years vs. 21 healthy controls, mean age 53 years). The GSE19322 dataset [14 healthy control mice vs. 16 AMI mice (1 day after AMI) vs. 16 AMI mice (5 days after AMI)], which describes the differences in the heart tissue of mice, was used to explore the dynamic changes in diagnostic markers. The scale function in R version 4.2.0 software was used to perform quality control and normalization of these two gene expression profiles, which are represented by boxplots. Principal component analysis (PCA) was used to verify the reproducibility of the data, and the R package ggord was used to construct the PCA plots. Figure 1 depicts the flow chart of this study.

### 2.2 Analysis of differentially expressed genes

Using the DAVID online database,^3^ gene probe IDs were converted to gene symbols using the “Gene ID Conversion” feature. Differential expression analysis was conducted using the “limma” package in R 4.2.0 software, and “ggplot2” and “ComplexHeatmap” were used to depict volcano/difference ranking plots and heatmap plots, respectively. GSEA was conducted using GSEA software (version 4.1.0) to clarify the potential mechanism in AMI. The DEGs were screened using the criteria of a |log2(FC)| > 1 and *p*-value < 0.05, and the enriched pathways of GSEA were screened using an FDR < 0.25 and a *p*-value < 0.05.

### 2.3 Analysis of differential expression of senescence-related genes

The “venneuler” package in R 4.2.0 software was adopted to draw the intersection of DEGs and SRGs, i.e., DESRGs. The KEGG pathway and GO enrichment of DESRGs were analyzed utilizing the “clusterProfiler” and “GOplot” packages of R software. An analysis of the PPI network of DESRGs was performed using the STRING database,^4^ and then the top 10 hub genes were obtained using the Degree algorithm of the Cytohubba plugin in Cytoscape software (version 3.9.0). The expression levels and Spearman correlations of DESRGs in the training set are displayed.

### 2.4 Identification of diagnostic genes

To screen the diagnostic genes, we visually displayed the expression levels of hub genes between AMI patients and healthy controls in the form of scatter plots and boxplots. ROC curve analysis was performed, and the AUCs were calculated using the pROC package in R software to determine the predicted values of the hub genes. Diagnostic genes were selected from the training set and validation set using the criterion of AUC > 0.700. To observe the dynamic changes in diagnostic genes in AMI, the expression levels and ROC curves of diagnostic genes in mice with AMI and healthy control mice were analyzed.

### 2.5 Construction of an miRNA-gene regulatory network

The miRNet database^5^ was applied to predict the interaction between diagnostic genes and miRNAs, and the miRNA-gene regulatory network was visualized using Cytoscape software.

### 2.6 Potential therapeutic drug prediction

The gene-drug interaction network was constructed to predict potential new targets for AMI drug synthesis using the Comparative Toxicogenomics Database (CTD) and was visualized with Cytoscape software. CTD manually collates associations among chemistry, genetics, phenotype and disease from the published literature and is widely used to predict potential drug targets for genes by integrating diverse data (13).

### 2.7 Statistical analysis

R 4.2.0 software and Adobe Photoshop 2021 were employed in this research. Data are presented as the mean ± SD, and comparisons between groups were performed using an unpaired Student’s *t*-test. ROCs were used to evaluate AUCs and predictive abilities. A *p*-value of less than 0.05 was considered statistically significant.

## 3 Results

### 3.1 Identification of DEGs and DESRGs in AMI circulating endothelial cells

**FIGURE 2:**
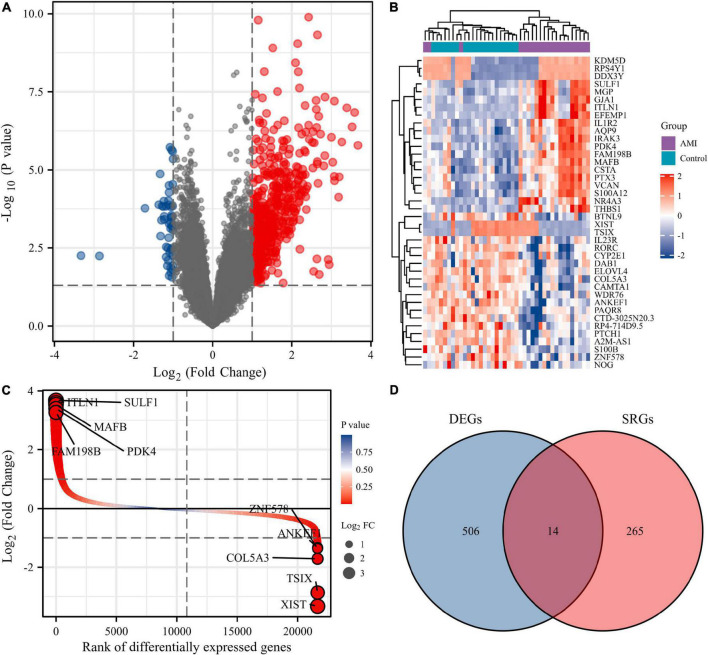
Identification of DEGs and DESRGs in AMI circulating endothelial cells in the GSE66360 training set. **(A)** Volcano plot, **(B)** heatmap, and **(C)** difference ranking plot of DEGs between the AMI and control samples. **(D)** Venn diagram of overlapping genes between the GSE66360 training set and CellAge database. DEGs, differentially expressed genes; SRGs, senescence-related genes.

**FIGURE 3:**
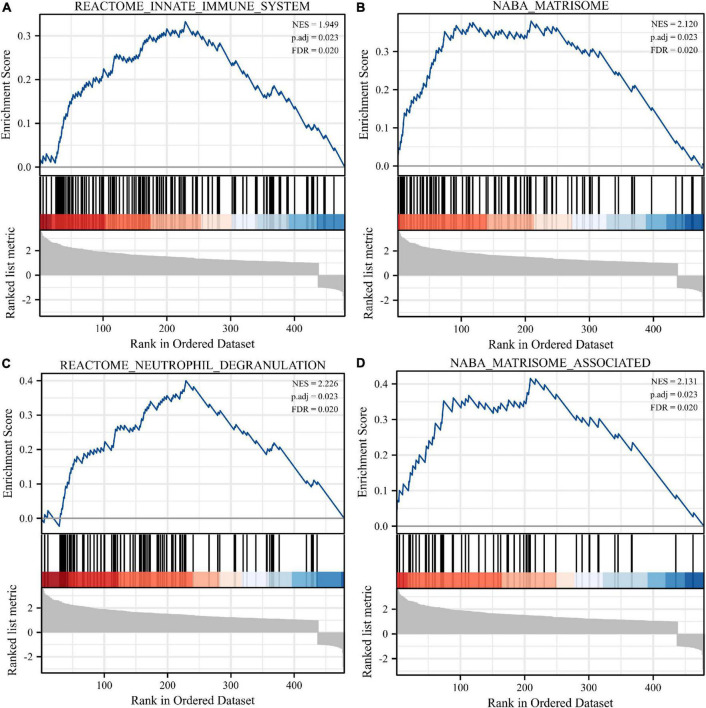
Enrichment analyses using gene set enrichment analysis (GSEA). Four significant gene set enrichment pathways (FDR < 0.25, adjusted *p* < 0.05). **(A)** Reactome innate immune system. **(B)** Naba matrisome. **(C)** Reactome neutrophil degranulation. **(D)** Naba matrisome associated. FDR, false discovery rate.

**FIGURE 4:**
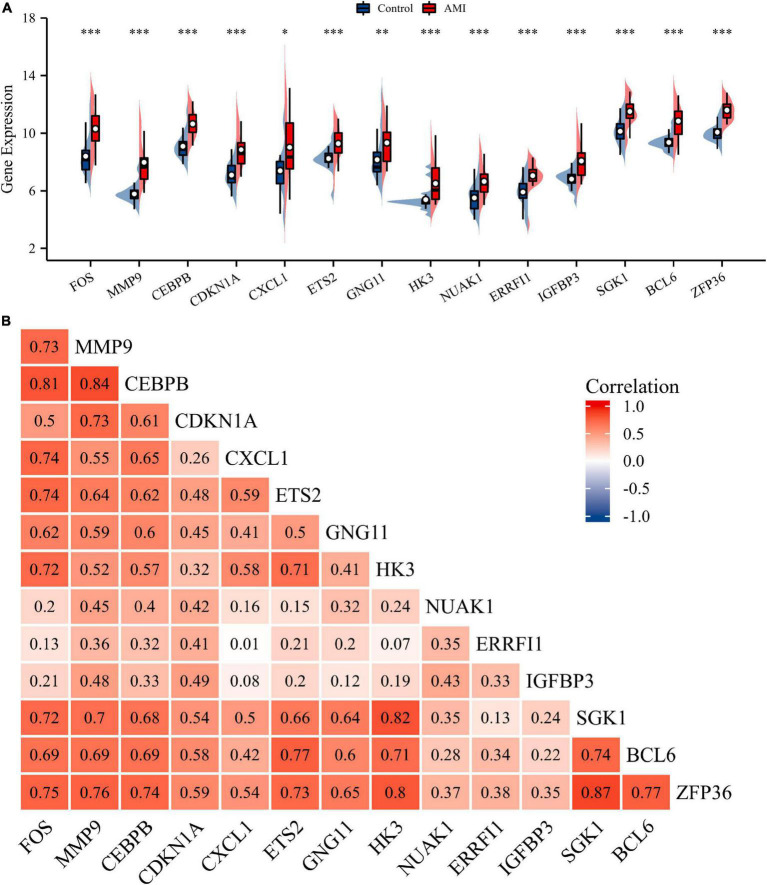
Expression and correlation analysis of 14 DESRGs. **(A)** Expression levels of DESRGs in the AMI and control samples in the GSE66360 training set were visualized by box plot. *p < 0.05, ^**^p < 0.01, ^***^p < 0.001. **(B)** Spearman correlation among 14 DESRGs. AMI, acute myocardial infarction; DESRGs, differentially expressed senescence-related genes.

Normalization was performed on the expression matrices of the GSE66360 (training set and validation set) and GSE48060 datasets, and the distribution trends of the boxplots were large straight lines (Supplementary Figure 1). The PCA revealed that the data were repeatable (Supplementary Figure 2). Using the thresholds of adjusted |log2(FC)| > 1 and *p*-value < 0.05, a total of 520 DEGs were obtained from the GSE66360 training set, including 471 significantly upregulated and 49 significantly downregulated genes (Supplementary Table 2). Volcano plots and heatmaps were used to visualize DEGs (Figures 2A,B), and the top five up- and downregulated genes were marked in the difference ranking plot (Figure 2C). The GSEA showed that DEGs were mainly involved in the Reactome innate immune system, Naba matrisome, Reactome neutrophil degranulation, and Naba matrisome associated (Figures 3A–D). Ultimately, combined with DEGs and SRGs, we screened 14 overlapping genes (DESRGs) for further study (Figure 2D), and all 14 DESRGs were upregulated. Figures 4A,B show the expression levels and correlations of the 14 DESRGs in the training set. Table 1 shows the information for the 14 DESRGs and their roles in senescence.

**TABLE 1:** Fourteen differentially expressed senescence-related genes (all upregulated genes).

Gene name	Description	Senescence effect
GNG11	G protein subunit gamma 11	Induces
CXCL1	C-X-C motif chemokine ligand 1	Induces
HK3	Hexokinase 3	Induces
ETS2	ETS proto-oncogene 2, transcription factor	Induces
ERRFI1	ERBB receptor feedback inhibitor 1	Induces
NUAK1	NUAK family kinase 1	Induces
IGFBP3	Insulin like growth factor binding protein 3	Induces
FOS	Fos proto-oncogene, AP-1 transcription factor subunit	Inhibits
SGK1	Serum/glucocorticoid regulated kinase 1	Inhibits
BCL6	B-cell CLL/lymphoma 6	Inhibits
CDKN1A	Cyclin dependent kinase inhibitor 1A	Induces
CEBPB	CCAAT/enhancer binding protein beta	Induces
MMP9	Matrix metallopeptidase 9	Inhibits
ZFP36	ZFP36 ring finger protein	Induces

### 3.2 Functional enrichment analysis of DESRGs

**FIGURE 5:**
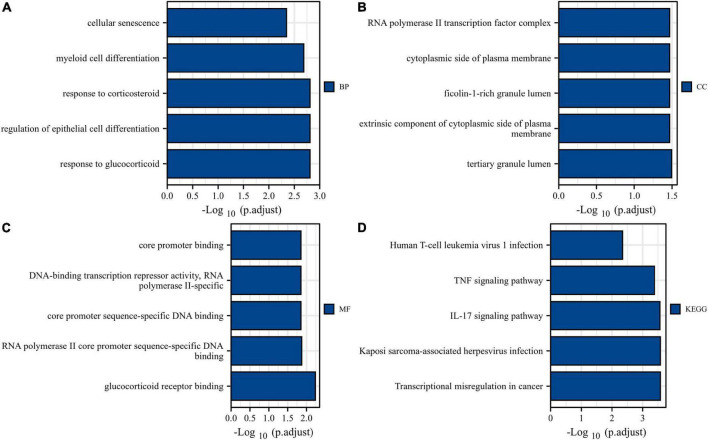
Bar plots of 14 DESRG-enriched GO terms and KEGG pathways. Panels **(A–D)** represent BP, CC, MF, and KEGG, respectively. GO, Gene Ontology; KEGG, Kyoto Encyclopedia of Genes and Genomes; BP, biological process; CC, cellular component; MF, molecular function; DESRGs, differentially expressed senescence-related genes.

**FIGURE 6:**
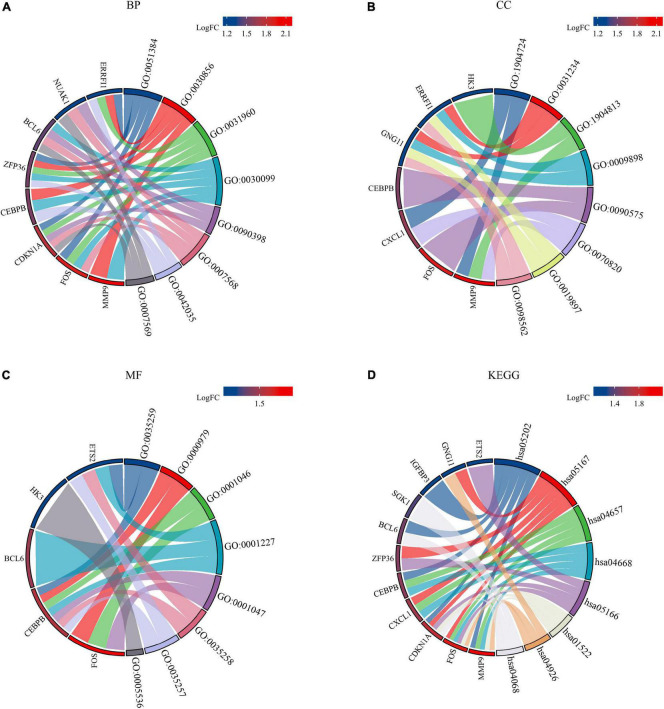
Chord plots of 14 DESRG-enriched GO terms and KEGG pathways. Panels **(A–D)** represent the top eight enriched items and their enriched genes of BP, CC, MF, and KEGG, respectively. GO, Gene Ontology; KEGG, Kyoto Encyclopedia of Genes and Genomes; BP, biological process; CC, cellular component; MF, molecular function; DESRGs, differentially expressed senescence-related genes.

Gene Ontology and KEGG analyses were conducted to reveal the possible biological functions and enrichment pathways of DESRGs. The GO analysis was categorized into biological processes (BPs), cell components (CCs), and molecular functions (MFs). The BP of DESRGs were mainly enriched in response to glucocorticoids, regulation of epithelial cell differentiation and response to corticosteroids. For CC, DESRGs were mainly enriched in the tertiary granule lumen, extrinsic component of the cytoplasmic side of the plasma membrane and ficolin-1-rich granule lumen. The DESRGs were enriched in MF, including glucocorticoid receptor binding, RNA polymerase II core promoter sequence-specific DNA binding and core promoter sequence-specific DNA binding. KEGG analysis showed that DESRGs were mainly associated with transcriptional misregulation in cancer, Kaposi sarcoma-associated herpesvirus infection and the IL-17 signaling pathway. Figures 5A–D show the top five enrichment items and (Figures 6A–D) show the enriched genes of BP, CC, MF, and KEGG.

### 3.3 Analysis of PPI networks and identification of hub genes

**FIGURE 7:**
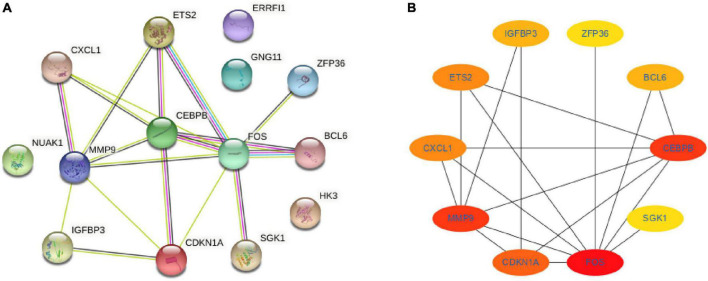
Protein-protein interaction network and hub genes. **(A)** Protein-protein interaction network constituted with the DESRGs; **(B)** top 10 hub genes. Darker colors indicate a higher value. DESRGs, differentially expressed senescence-related genes.

The STRING database was used to construct the PPI network to identify the interactive relationships between DESRGs. A total of 14 nodes and 18 edges were identified in the PPI network (Figure 7A). The Cytohubba plug-in in Cytoscape software was then used to select the top 10 hub genes based on their degree of connectivity (Figure 7B).

### 3.4 Identification and validation of diagnostic feature biomarkers

**FIGURE 8:**
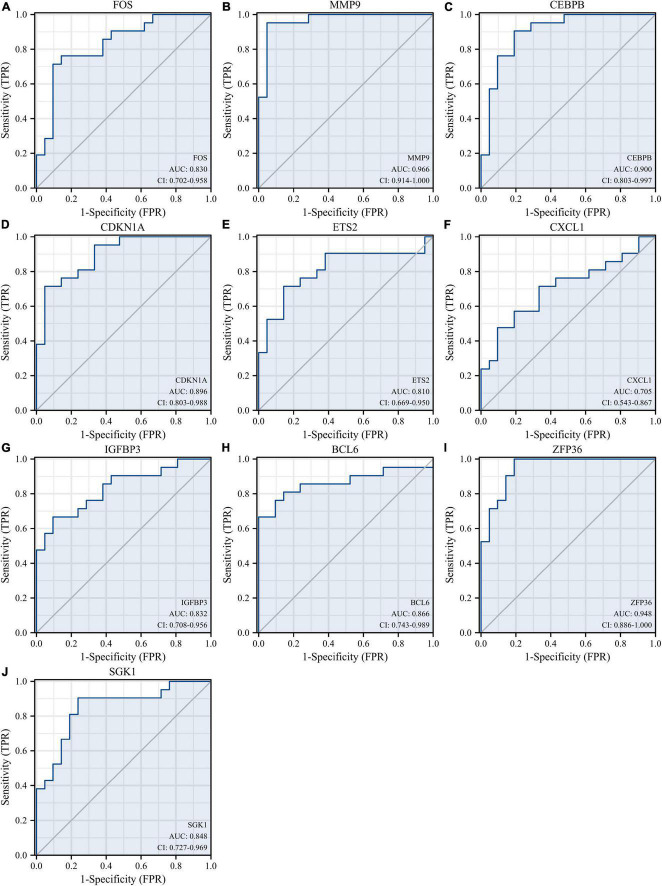
Diagnostic value of 10 hub genes in the GSE66360 training set. ROC curves of FOS **(A)**, MMP9 **(B)**, CEBPB **(C)**, CDKN1A **(D)**, ETS2 **(E)**, CXCL1 **(F)**, IGFBP3 **(G)**, BCL6 **(H)**, ZFP36 **(I)**, and SGK1 **(J)**. ROC, receiver operating characteristic.

**FIGURE 9:**
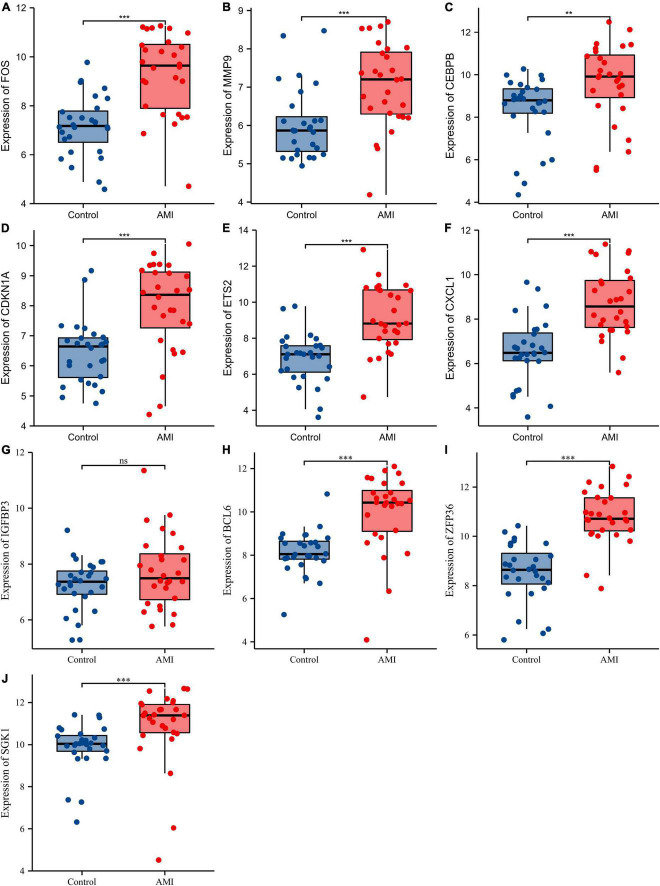
Comparison of the expression of 10 hub genes in the GSE66360 validation set. Expression levels of FOS **(A)**, MMP9 **(B)**, CEBPB **(C)**, CDKN1A **(D)**, ETS2 **(E)**, CXCL1 **(F)**, IGFBP3 **(G)**, BCL6 **(H)**, ZFP36 **(I)**, and SGK1 **(J)**. ns, no significance; ^**^p < 0.01; ^***^p < 0.001.

**FIGURE 10:**
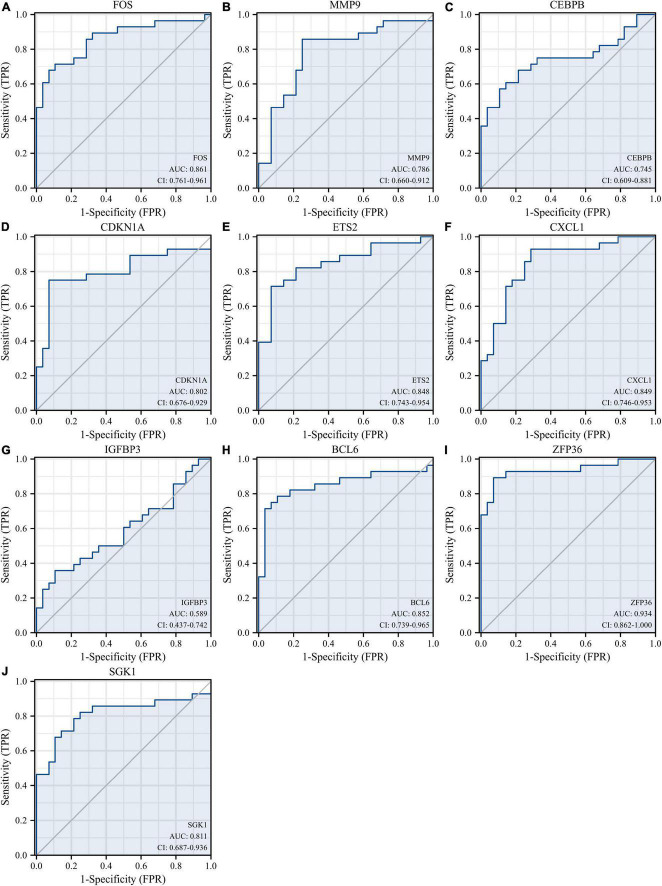
Diagnostic values of 10 hub genes in the GSE66360 validation set. ROC curves of FOS **(A)**, MMP9 **(B)**, CEBPB **(C)**, CDKN1A **(D)**, ETS2 **(E)**, CXCL1 **(F)**, IGFBP3 **(G)**, BCL6 **(H)**, ZFP36 **(I)**, and SGK1 **(J)**. ROC, receiver operating characteristic.

**FIGURE 11:**
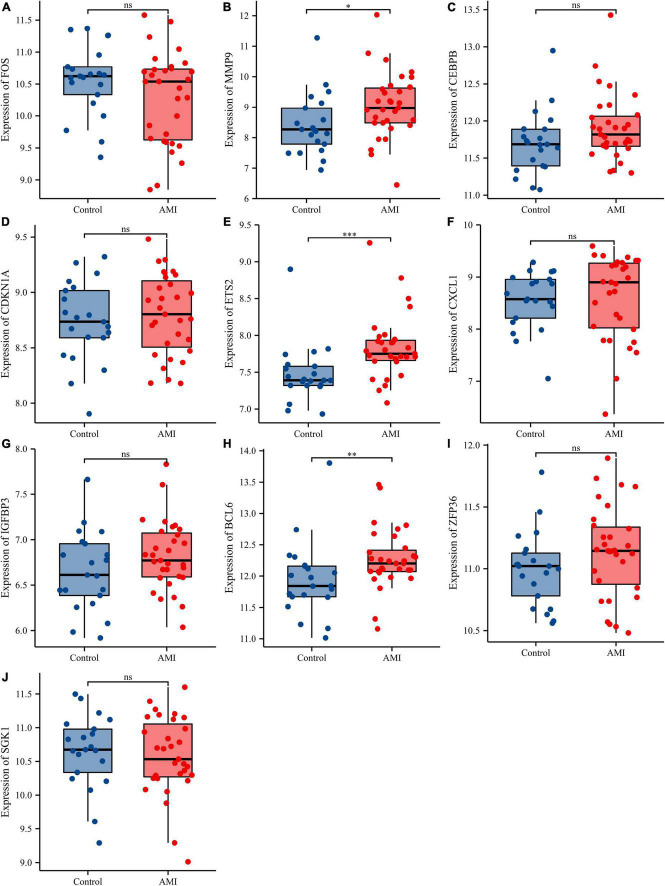
Comparison of the expressions of 10 hub genes in the GSE48060 dataset. Expression levels of FOS **(A)**, MMP9 **(B)**, CEBPB **(C)**, CDKN1A **(D)**, ETS2 **(E)**, CXCL1 **(F)**, IGFBP3 **(G)**, BCL6 **(H)**, ZFP36 **(I)**, and SGK1 **(J)**. ns, no significance; *p < 0.05; ^**^p < 0.01; ^***^p < 0.001.

**FIGURE 12:**
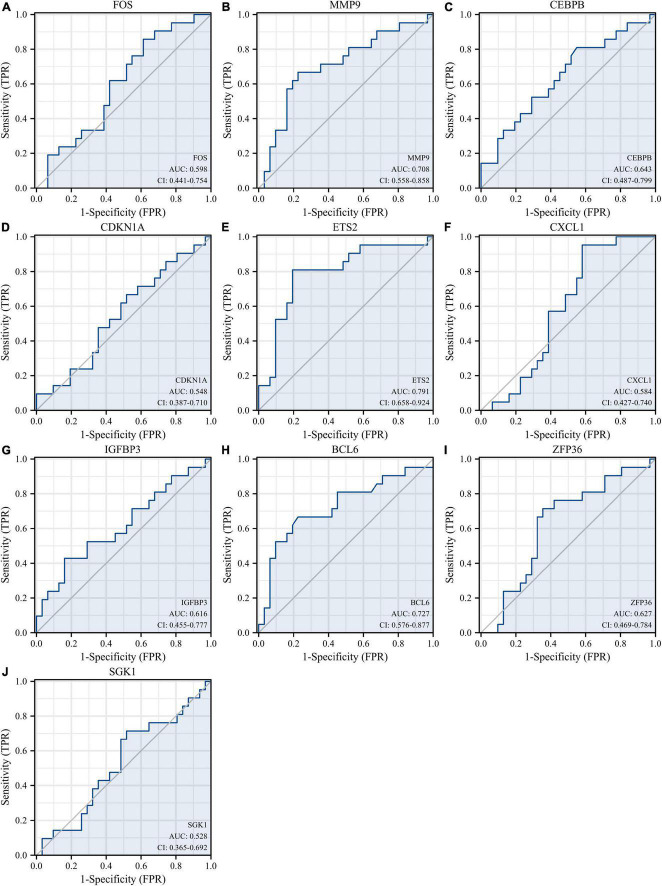
Diagnostic values of 10 hub genes in the GSE48060 dataset. ROC curves of FOS **(A)**, MMP9 **(B)**, CEBPB **(C)**, CDKN1A **(D)**, ETS2 **(E)**, CXCL1 **(F)**, IGFBP3 **(G)**, BCL6 **(H)**, ZFP36 **(I)**, and SGK1 **(J)**. ROC, receiver operating characteristic.

**FIGURE 13:**
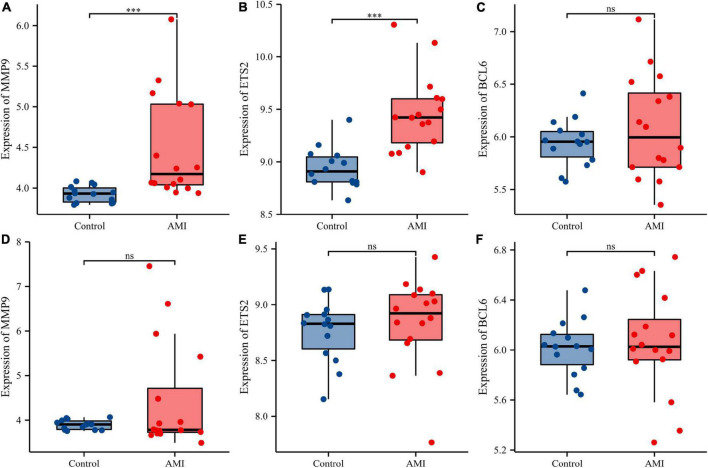
Comparison of the expressions of three diagnostic genes in the GSE19322 dataset. Expression levels of MMP9 **(A)**, ETS2 **(B)**, and BCL6 **(C)** at 1 day after AMI; expression levels of MMP9 **(D)**, ETS2 **(E)**, and BCL6 **(F)** at 5 days after AMI. ns, no significance; ^***^p < 0.001.

**FIGURE 14:**
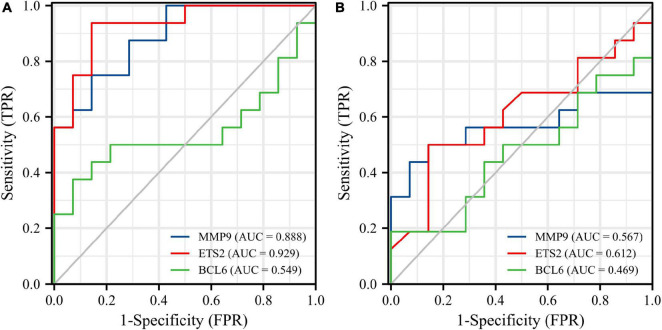
Diagnostic values of three diagnostic genes in the GSE19322 dataset. ROC curves of MMP9 (blue), ETS2 (red), and BCL6 (green) at 1 day after AMI **(A)**. ROC curves of MMP9 (blue), ETS2 (red), and BCL6 (green) at 5 days after AMI **(B)**. ROC, receiver operating characteristic.

Receiver operating characteristic curves were used to evaluate the diagnostic value of 10 hub genes in AMI. Figures 8A–J show the diagnostic values of the 10 hub genes in the training set. The results showed that the AUCs of all hub genes were greater than 0.800, among which MMP9 (AUC, 0.996) had the highest diagnostic value, followed by ZFP36 (AUC, 0.948). In the validation set, the gene expression level and diagnostic value were further verified. Figures 9A–J, 10A–J show the gene expression levels and diagnostic values in the GSE66360 validation set. The gene expression levels between the AMI group and the control group were statistically significant. Except IGFBP33, the AUCs of all hub genes were greater than 0.700, of which ZFP36 (AUC, 0.934) had the highest diagnostic value, followed by FOS (AUC, 0.861). However, in the GSE48060 datasets, only the expression levels of ETS2 (AUC, 0.791), BCL6 (AUC, 0.727) and MMP9 (AUC, 0.708) were statistically significant, and their AUCs exceeded 0.700, showing good diagnostic value (Figures 11A–J, 12A–J). Finally, we screened the overlapping genes in the training set and the validation set as diagnostic genes: ETS2, BCL6, and MMP9 (AUC > 0.700). Figures 13A–F show the expression levels of MMP9, ETS2, and BCL6 at 1 and 5 days, respectively, after AMI in the GSE19322 dataset. Figures 14A,B show the diagnostic values of MMP9, ETS2, and BCL6 at 1 and 5 days, respectively, after AMI in the GSE19322 dataset. The results showed that the expressions of MMP9 (AUC, 0.888) and ETS2 (AUC, 0.929) had statistical significance and high diagnostic value in the early stage of AMI.

### 3.5 Construction of the miRNA-gene network and potential therapeutic drug prediction

**FIGURE 15:**
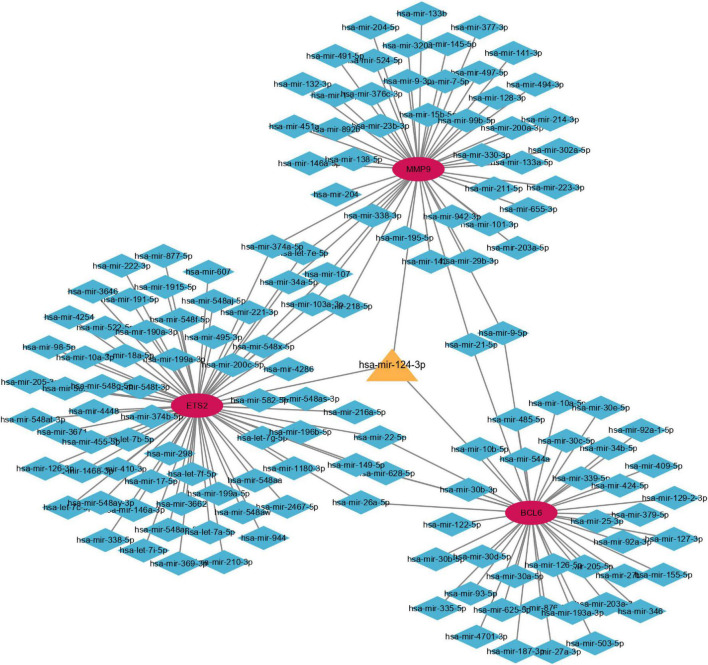
Coexpression network of diagnostic genes and target miRNAs. Red ellipses represent diagnostic genes, and blue diamonds represent target miRNAs.

The miRNet tool was used to predict target microRNAs (miRNAs) of the diagnostic genes (ETS2, BCL6, and MMP9). Ultimately, we obtained 148 miRNAs of 3 diagnostic genes (Supplementary Table 3). ETS2 was regulated by 68 miRNAs, BCL6 was regulated by 43 miRNAs and MMP9 was regulated by 48 miRNAs, and these three genes were simultaneously regulated by hsa-mir-124-3p. The miRNA-gene network, which comprised 148 nodes and 159 edges, was constructed using Cytoscape (Figure 15). As shown in Supplementary Figure 3, multiple drugs may affect the expression of these three diagnostic genes in AMI. There were 108 interacting drugs for MMP9, 34 interacting drugs for BCL6, and 28 interacting drugs for ETS2. Finally, based on the potential drugs of the three diagnostic genes of AMI, we obtained 10 overlapping drugs as target drugs closely related to cellular senescence, including acetylcysteine, bisphenol A, bleomycin, cisplatin, clofibrate, cobaltous chloride, dexamethasone, genistein, tamoxifen, and tretinoin; their chemical structures are shown in Supplementary Figure 4.

## 4 Discussion

Acute myocardial infarction is one of the leading causes of death worldwide and can cause irreversible damage to myocardial tissue (14). Epidemiological studies have confirmed that aging is a major risk factor for atherosclerosis (10). Cellular senescence, which refers to the irreversible stagnation of cell division and proliferation, is a hallmark of aging and is associated with aging-related diseases (15). Endothelial cell senescence is thought to play an important role in aging-related organ dysfunction (11). The search for efficient biomarkers representing cellular senescence has attracted widespread attention, but the diagnostic biomarkers of cellular senescence in AMI remain unexplored. Therefore, this study focused on using bioinformatics to screen cellular senescence-related diagnostic biomarkers in the circulating endothelial cells of AMI patients.

Recently, several studies have focused on the analysis of DEGs in AMI patients. For example, Zhao et al. predicted diagnostic genetic biomarkers associated with immune infiltration in patients with AMI (16). Xue et al. used weighted gene coexpression network analysis to identify and validate hub genes associated with AMI (17). However, bioinformatic analysis of SRGs in AMI has not yet been performed. In the present study, 520 DEGs and 279 SRGs were used to identify 14 DESRGs, including 4 genes that inhibited and 10 genes that induced senescence effects (Table 1). Three diagnostic biomarkers were identified: MMP9, ETS2, and BCL6.

Matrix metalloproteinase 9 (MMP9), is a member of the zinc ion-dependent protease family and is a gelatinase secreted by leukocytes, fibroblasts, macrophages, epithelial cells, and endothelial cells (18–20). MMP9 both selectively degrades different extracellular matrix (ECM) components (18) and processes a variety of cytokines, growth factors, and other matrix metalloproteinases (21). Numerous studies have confirmed the role of MMP9 in cardiac aging. MMP9 activity increases with age in both spontaneously hypertensive and normotensive rats (22). MMP9 depletion attenuates aging-related cardiac fibrosis and diastolic dysfunction by mediating inflammatory responses to aging (23). It also stimulates anti-inflammatory polarization of macrophages to alleviate left ventricular dysfunction in post-aging myocardial infarction (24). MMP9 and its inhibitors can be considered new predictors of dilated cardiomyopathy and used to evaluate treatment effectiveness in patients of different ages (25). Furthermore, MMP9 has been identified as one of the hub genes or diagnostic markers of AMI by many studies using bioinformatics approaches (26–28). Notably, none of the studies investigated the role of MMP9 in cellular senescence and AMI. Therefore, by exploring the relationship between aging and AMI, we found that expression of the SRG MMP9 was significantly increased in AMI patients, and further studies showed that MMP9 could be used as a diagnostic marker in AMI.

ETS2, an ETS proto-oncogene 2 transcription factor, is a member of the ETS family of transcription factors that regulates macrophages during inflammation and participates in the regulation of tumor-associated macrophages (29). ETS2 has not only been shown to be a potent transactivator of angiogenesis regulators but also participates in the transcriptional regulation of cytokines in immune activation (30, 31). The vast majority of research on ETS2 has focused on tumors such as breast cancer (32) and non-small cell lung cancers (33). Studies have shown that overexpression of ETS2 promotes neovascularization, hemorrhaging, and plaque destabilization in a murine vulnerable plaque model (31). This study observed an association between elevated ETS2 levels within plaques and atherogenic inflammatory markers, including tumor necrosis factor (TNF) and interleukin 6 (IL6). Therefore, it was hypothesized that ETS2 may have a proatherogenic function in the development of advanced atherosclerotic plaques. ETS2 regulates the expression of miR-126, which is highly expressed in endothelial cells and regulates angiogenesis and vascular inflammation (34). In surviving mice with myocardial infarction, targeted deletion of miR-126 resulted in increased mortality (35). In addition, ETS2 was confirmed to be a key transcription factor for the induction of miR-155 in the inflammatory response in lipopolysaccharide-activated macrophage mice (36). miR-155 is highly expressed in inflammatory diseases and exhibits powerful proinflammatory activity (37). A decrease in EST2 expression has also been described in senescent cells (38). Our study showed that the SRG ETS2 was significantly elevated in AMI patients. Based on the above findings, we hypothesized that ETS2 mainly plays an inflammatory role in AMI, and this role is mainly achieved by mediating certain microRNAs, possibly miR-126 and miR-155, or others. However, more research is needed to confirm this hypothesis.

BCL6, BCL6 transcription repressor or B-cell CLL/lymphoma 6, is a sequence-specific DNA-binding protein that inhibits transcription through interactions with various corepressors (39) and has been extensively studied in autoimmune diseases and cancer (40). BCL6 also plays a very important role in the cardiovascular system. Expression and genomic analysis have identified SMRT and NCoR as the major components of BCL6 against atherogenic and NF-κB-driven inflammation (41). Those results suggest that BCL6-SMRT/NCoR complexes suppress immune responses and contribute to the prevention of atherosclerosis. In AMI, BCL6 has also been reported, and mesenchymal stem cell-derived extracellular vesicles carrying miR-302D-3p can inhibit inflammation and cardiac remodeling after AMI *via* the BCL6/MD2/NF-κB axis (42). BCL6 is involved in the regulation of cardiomyocyte activation and function, and cardiomyocyte hypoxic injury and cardiomyocyte apoptosis have also been reported (43–45). Moreover, Altieri et al. found that BCL6 cooperates with PPARdelta to protect the heart from doxorubicin-induced senescence (46). Using bioinformatics, the circRNA-miRNA-mRNA network constructed by Zhou et al. for AMI suggested that the hsa_circ_0009018/hsa-miR-139-3p/BCL6 axis was significantly positively correlated with upregulated immune cells in AMI, which may be represent potential therapeutic targets (47). All of these data suggest the importance of BCL6 in cardiovascular disease or senescence. Our study showed that the SRG BCL6 could be used as a biomarker for the diagnosis of AMI.

Numerous studies have shown that the abnormal expression of miRNAs is related to the pathological process of AMI (48, 49). In AMI rats, inhibiting the expression of miR-124-3p can reduce apoptosis, inflammation and oxidative stress (50). In the blood of patients with AMI and hypoxia-treated H9c2 cells, the expression of miR-124-3p was significantly increased (51). Further studies have demonstrated that downregulation of miR-124-3p can inhibit inflammation and cardiomyocyte apoptosis by regulating the activated B-cell suppressor factor (NKRF)/NF-κB pathway to prevent AMI (51). Several miRNAs such as miR-17-3p, miR-34a, and miR-22 are reported to be involved in the pathophysiology of cell senescence (52). However, to the best of our knowledge, the association between miR-124-3p and cell senescence has not been reported. The coexpression network of diagnostic genes and target miRNAs constructed in our study suggests that miR-124-3p, as a common target miRNA of MMP9, ETS2, and BCL6, may play a role in the pathological process of AMI and may be a new potential target for the prevention and treatment of AMI. Finally, targeted drugs for the treatment of senescence-related AMI were also explored in this study. N-acetylcysteine and genistein have been reported to play a protective role in cellular senescence (53, 54). Bisphenol A, bleomycin, cisplatin and dexamethasone have been reported to induce cell senescence under some special circumstances (55–58). Moreover, conventional drugs that improve cardiac function after AMI such as the calcium sensitizer levosimendan and the angiotensin II-receptor blocker losartan have been found to reduce the expression of markers of senescence in heart tissue (52). However, further studies are needed to determine whether age-related AMI would benefit from the use of these target drugs. In future studies, we will focus on exploring the relationship between miR-124-3p and cellular senescence and further investigate the mechanisms involved in the induction and protection against cellular senescence by these drugs.

There are some limitations in our research that should be noted. First, our research only selected cellular SRGs from the CellAge database. In fact, there should be more cellular SRGs that have not been identified. Second, our sample sizes were relatively small, particularly for the training set, although we performed similar internal and external validations to compensate for this limitation. Third, failure to find human gene expression profiles that could be used to explore the dynamic evolution of the screened diagnostic genes in AMI may lead to different results. Finally, although the three diagnostic genes we screened have been reported to be associated with cellular senescence, there are insufficient studies to confirm the role of senescence regulated by these genes in AMI. Therefore, more *in vitro* and *in vivo* studies are required to confirm these findings.

## 5 Conclusion

In conclusion, we identified and validated three SRGs (MMP9, ETS2, and BCL6) as novel diagnostic biomarkers in the early stages of AMI. These genes may participate in the occurrence and development of AMI through the inflammatory response or immune regulation. Our findings may provide potential targets for the treatment of AMI.

## Data availability statement

The original contributions presented in this study are included in the article/Supplementary material, further inquiries can be directed to the corresponding authors.

## Author contributions

JX and JS: design, methodology, software application, data curation, and manuscript writing. LZ and BT: visualization, investigation, and validation. BT: supervision and writing—reviewing and editing. All authors contributed to the article and approved the submitted version.

## Footnotes

1
https://www.ncbi.nlm.nih.gov/geo/

2
https://genomics.senescence.info/

3
http://david.ncifcrf.gov/

4
https://string-db.org/

5
https://www.mirnet.ca/

## Supplementary material

Supplementary Figure 1 Distribution trends for box plots of datasets.

Supplementary Figure 2 Principal component analysis of datasets.

Supplementary Figure 3 Gene-drug interaction network constructed with three diagnostic genes and therapeutic drugs. Red ellipses represent diagnostic genes, and blue diamonds represent target drugs.

Supplementary Figure 4 The chemical structures of the therapeutic target drugs. **(A)** Acetylcysteine, **(B)** tretinoin, **(C)** clofibrate, **(D)** bleomycin, **(E)** bisphenol A, **(F)** dexamethasone, **(G)** tamoxifen, **(H)** cobaltous chloride, **(I)** cisplatin, and **(J)** genistein.

Supplementary Table 1 Senescence-related genes.

Supplementary Table 2 Significantly up- and downregulated genes.

Supplementary Table 3 One hundred and forty-eight miRNAs of 3 diagnostic genes.

## References

[1] Hong G Rui G Zhang D Lian M Yang Y Chen P A smartphone-assisted pressure-measuring-based diagnosis system for acute myocardial infarction diagnosis. *Int J Nanomedicine.* (2019). 14:2451–64. 10.2147/IJN.S197541 31040668 PMC6459154

[2] Singhanat K Apaijai N Jaiwongkam T Kerdphoo S Chattipakorn SC Chattipakorn N. Melatonin as a therapy in cardiac ischemia-reperfusion injury: potential mechanisms by which MT2 activation mediates cardioprotection. *J Adv Res.* (2020) 29:33–44. 10.1016/j.jare.2020.09.007 33842003 PMC8020169

[3] Twerenbold R Jaeger C Rubini Gimenez M Wildi K Reichlin T Nestelberger T Impact of high-sensitivity cardiac troponin on use of coronary angiography, cardiac stress testing, and time to discharge in suspected acute myocardial infarction. *Eur Heart J.* (2016) 37:3324–32. 10.1093/eurheartj/ehw232 27357358 PMC5177796

[4] Van de Werf F Bax J Betriu A Blomstrom-Lundqvist C Crea F Falk V Management of acute myocardial infarction in patients presenting with persistent ST-segment elevation: the task force on the management of ST-Segment elevation acute myocardial infarction of the European Society of Cardiology. *Eur Heart J.* (2008) 29:2909–45. 10.1093/eurheartj/ehn416 19004841

[5] Lieder HR Kleinbongard P Skyschally A Hagelschuer H Chilian WM Heusch G. Vago-Splenic axis in signal transduction of remote ischemic preconditioning in pigs and rats. *Circ Res.* (2018) 123:1152–63. 10.1161/CIRCRESAHA.118.313859 30359199 PMC7304918

[6] Bhatia S Arora S Bhatia SM Al-Hijji M Reddy YNV Patel P Non-ST-Segment-Elevation myocardial infarction among patients with chronic kidney disease: a propensity score-matched comparison of percutaneous coronary intervention versus conservative management. *J Am Heart Assoc.* (2018) 7:e007920. 10.1161/JAHA.117.007920 29525779 PMC5907556

[7] Cohen M Gensini GF Maritz F Gurfinkel EP Huber K Timerman A Prospective evaluation of clinical outcomes after acute ST-elevation myocardial infarction in patients who are ineligible for reperfusion therapy: preliminary results from the TETAMI registry and randomized trial. *Circulation.* (2003) 108(16 Suppl. 1):III14–21. 10.1161/01.CIR.0000091832.74006.1C 14605015

[8] Alique M Luna C Carracedo J Ramírez R. LDL biochemical modifications: a link between atherosclerosis and aging. *Food Nutr Res.* (2015) 59:29240. 10.3402/fnr.v59.29240 26637360 PMC4670441

[9] Le Blanc J Lordkipanidzé M. Platelet function in aging. *Front Cardiovasc Med.* (2019) 6:109. 10.3389/fcvm.2019.00109 31448291 PMC6692461

[10] Tyrrell DJ Blin MG Song J Wood SC Zhang M Beard DA Age-Associated mitochondrial dysfunction accelerates atherogenesis. *Circ Res.* (2020) 126:298–314. 10.1161/CIRCRESAHA.119.315644 31818196 PMC7006722

[11] Barinda AJ Ikeda K Nugroho DB Wardhana DA Sasaki N Honda S Endothelial progeria induces adipose tissue senescence and impairs insulin sensitivity through senescence associated secretory phenotype. *Nat Commun.* (2020) 11:481. 10.1038/s41467-020-14387-w 31980643 PMC6981212

[12] Zhang Y Herbert BS Rajashekhar G Ingram DA Yoder MC Clauss M Premature senescence of highly proliferative endothelial progenitor cells is induced by tumor necrosis factor-alpha via the p38 mitogen-activated protein kinase pathway. *FASEB J.* (2009) 23:1358–65. 10.1096/fj.08-110296 19124561 PMC2669419

[13] Davis AP Wiegers TC Johnson RJ Sciaky D Wiegers J Mattingly CJ. Comparative Toxicogenomics Database (CTD): update 2023. *Nucleic Acids Res.* (2022) [Epub ahead of print]. 10.1093/nar/gkac833 36169237 PMC9825590

[14] Thygesen K Alpert JS Jaffe AS Chaitman BR Bax JJ Morrow DA Fourth universal definition of myocardial infarction (2018). *J Am Coll Cardiol.* (2018) 72:2231–64. 30153967 10.1016/j.jacc.2018.08.1038

[15] Cai Y Zhou H Zhu Y Sun Q Ji Y Xue A Elimination of senescent cells by β-galactosidase-targeted prodrug attenuates inflammation and restores physical function in aged mice. *Cell Res.* (2020) 30:574–89. 10.1038/s41422-020-0314-9 32341413 PMC7184167

[16] Zhao E Xie H Zhang Y. Predicting diagnostic gene biomarkers associated with immune infiltration in patients with acute myocardial infarction. *Front Cardiovasc Med.* (2020) 7:586871. 10.3389/fcvm.2020.586871 33195475 PMC7644926

[17] Xue J Chen L Cheng H Song X Shi Y Li L The identification and validation of hub genes associated with acute myocardial infarction using weighted gene co-expression network analysis. *J Cardiovasc Dev Dis.* (2022) 9:30. 10.3390/jcdd9010030 35050240 PMC8778825

[18] Hane H Muro Y Watanabe K Ogawa Y Sugiura K Akiyama M. Establishment of an ELISA to detect anti-glycyl-tRNA synthetase antibody (anti-EJ), a serological marker of dermatomyositis/polymyositis and interstitial lung disease. *Clin Chim Acta.* (2014) 431:9–14. 10.1016/j.cca.2014.01.005 24508626

[19] Liu Y Luo H Wang L Li C Liu L Huang L Increased serum matrix metalloproteinase-9 levels are associated with Anti-Jo1 but not Anti-MDA5 in myositis patients. *Aging Dis.* (2019) 10:746–55. 10.14336/AD.2018.1120 31440381 PMC6675534

[20] Creemers EE Cleutjens JP Smits JF Daemen MJ. Matrix metalloproteinase inhibition after myocardial infarction: a new approach to prevent heart failure? *Circ Res.* (2001) 89:201–10. 10.1161/hh1501.094396 11485970

[21] Meschiari CA Ero OK Pan H Finkel T Lindsey ML. The impact of aging on cardiac extracellular matrix. *Geroscience.* (2017) 39:7–18. 10.1007/s11357-017-9959-9 28299638 PMC5352584

[22] Kollarova M Puzserova A Balis P Radosinska D Tothova L Bartekova M Age- and phenotype-dependent changes in circulating MMP-2 and MMP-9 activities in normotensive and hypertensive rats. *Int J Mol Sci.* (2020) 21:7286. 10.3390/ijms21197286 33023122 PMC7582756

[23] Ma Y Chiao YA Clark R Flynn ER Yabluchanskiy A Ghasemi O Deriving a cardiac ageing signature to reveal MMP-9-dependent inflammatory signalling in senescence. *Cardiovasc Res.* (2015) 106:421–31. 10.1093/cvr/cvv128 25883218 PMC4498140

[24] Yabluchanskiy A Ma Y DeLeon-Pennell KY Altara R Halade GV Voorhees AP Myocardial infarction superimposed on aging: MMP-9 deletion promotes M2 macrophage polarization. *J Gerontol A Biol Sci Med Sci.* (2016) 71:475–83. 10.1093/gerona/glv034 25878031 PMC5175450

[25] Antonov IB Kozlov KL Pal’tseva EM Shanthi Pillai MR. Matrix Metalloproteinases MMP-1 and MMP-9 and their Inhibitor TIMP-1 as markers of dilated cardiomyopathy in patients of different age. *Bull Exp Biol Med.* (2018) 164:550–3. 10.1007/s10517-018-4030-0 29504111

[26] Yu YW Xue YJ Qian LL Chen Z Que JQ Huang KY Screening and identification of potential hub genes in myocardial infarction through bioinformatics analysis. *Clin Interv Aging.* (2020) 15:2233–43. 10.2147/CIA.S281290 33293800 PMC7718865

[27] Feng S Li R Zhou Q Qu F Hu W Liu X. Bioinformatics analysis to identify potential biomarkers and therapeutic targets for ST-segment-elevation myocardial infarction-related ischemic stroke. *Front Neurol.* (2022) 13:894289. 10.3389/fneur.2022.894289 36034287 PMC9403764

[28] Wu Y Jiang T Hua J Xiong Z Chen H Li L Integrated bioinformatics-based analysis of hub genes and the mechanism of immune infiltration associated with acute myocardial infarction. *Front Cardiovasc Med.* (2022) 9:831605. 10.3389/fcvm.2022.831605 35463752 PMC9019083

[29] Zabuawala T Taffany DA Sharma SM Merchant A Adair B Srinivasan R An ets2-driven transcriptional program in tumor-associated macrophages promotes tumor metastasis. *Cancer Res.* (2010) 70:1323–33. 10.1158/0008-5472.CAN-09-1474 20145133 PMC2822898

[30] Wakiya K Begue A Stehelin D Shibuya M. A cAMP response element and an Ets motif are involved in the transcriptional regulation of flt-1 tyrosine kinase (vascular endothelial growth factor receptor 1) gene. *J Biol Chem.* (1996) 271:30823–8. 10.1074/jbc.271.48.30823 8940064

[31] Cheng C Tempel D Den Dekker WK Haasdijk R Chrifi I Bos FL ETS2 determines the inflammatory state of endothelial cells in advanced atherosclerotic lesions. *Circ Res.* (2011) 109:382–95. 10.1161/CIRCRESAHA.111.243444 21700929

[32] Turner DP Findlay VJ Moussa O Watson DK. Defining ETS transcription regulatory networks and their contribution to breast cancer progression. *J Cell Biochem.* (2007) 102:549–59. 10.1002/jcb.21494 17661355

[33] Kabbout M Garcia MM Fujimoto J Liu DD Woods D Chow CW ETS2 mediated tumor suppressive function and MET oncogene inhibition in human non-small cell lung cancer. *Clin Cancer Res.* (2013) 19:3383–95. 10.1158/1078-0432.CCR-13-0341 23659968 PMC3846434

[34] Harris TA Yamakuchi M Kondo M Oettgen P Lowenstein CJ. ETS-1 and ETS-2 regulate the expression of microRNA-126 in endothelial cells. *Arterioscler Thromb Vasc Biol.* (2010) 30:1990–7. 10.1161/ATVBAHA.110.211706 20671229 PMC3121560

[35] Wang S Aurora AB Johnson BA Qi X McAnally J Hill JA The endothelial-specific microRNA miR-126 governs vascular integrity and angiogenesis. *Dev Cell.* (2008) 15:261–71. 10.1016/j.devcel.2008.07.002 18694565 PMC2685763

[36] Quinn SR Mangan NE Caffrey BE Gantier MP Williams BR Hertzog PJ The role of Ets2 transcription factor in the induction of microRNA-155 (miR-155) by lipopolysaccharide and its targeting by interleukin-10. *J Biol Chem.* (2014) 289:4316–25. 10.1074/jbc.M113.522730 24362029 PMC3924294

[37] Bala S Csak T Saha B Zatsiorsky J Kodys K Catalano D The pro-inflammatory effects of miR-155 promote liver fibrosis and alcohol-induced steatohepatitis. *J Hepatol.* (2016) 64:1378–87. 10.1016/j.jhep.2016.01.035 26867493 PMC4874886

[38] Ohtani N Zebedee Z Huot TJ Stinson JA Sugimoto M Ohashi Y Opposing effects of Ets and Id proteins on p16INK4a expression during cellular senescence. *Nature.* (2001) 409:1067–70. 10.1038/35059131 11234019

[39] Basso K Dalla-Favera R. BCL6: master regulator of the germinal center reaction and key oncogene in B cell lymphomagenesis. *Adv Immunol.* (2010) 105:193–210. 10.1016/S0065-2776(10)05007-8 20510734

[40] Cardenas MG Oswald E Yu W Xue F MacKerell AD Jr Melnick AM The expanding role of the bcl6 oncoprotein as a cancer therapeutic target. *Clin Cancer Res.* (2017) 23:885–93. 10.1158/1078-0432.CCR-16-2071 27881582 PMC5315622

[41] Barish GD Yu RT Karunasiri MS Becerra D Kim J Tseng TW The Bcl6-SMRT/NCoR cistrome represses inflammation to attenuate atherosclerosis. *Cell Metab.* (2012) 15:554–62. 10.1016/j.cmet.2012.02.012 22465074 PMC3367511

[42] Liu Y Guan R Yan J Zhu Y Sun S Qu Y Mesenchymal stem cell-derived extracellular vesicle-shuttled microRNA-302d-3p represses inflammation and cardiac remodeling following acute myocardial infarction. *J Cardiovasc Transl Res.* (2022) 15:754–71. 10.1007/s12265-021-10200-1 35194734

[43] Ni J Wu QQ Liao HH Fan D Tang QZ. Bcl6 suppresses cardiac fibroblast activation and function via directly binding to Smad4. *Curr Med Sci.* (2019) 39:534–40. 10.1007/s11596-019-2070-y 31346987

[44] Gu Y Luo M Li Y Su Z Wang Y Chen X Bcl6 knockdown aggravates hypoxia injury in cardiomyocytes via the P38 pathway. *Cell Biol Int.* (2019) 43:108–16. 10.1002/cbin.11028 29972264 PMC13397402

[45] Lin JM Hsu CH Chen JC Kao SH Lin YC. BCL-6 promotes the methylation of miR-34a by recruiting EZH2 and upregulating CTRP9 to protect ischemic myocardial injury. *Biofactors.* (2021) 47:386–402. 10.1002/biof.1704 33502806

[46] Altieri P Spallarossa P Barisione C Garibaldi S Garuti A Fabbi P Inhibition of doxorubicin-induced senescence by PPARδ activation agonists in cardiac muscle cells: cooperation between PPARδ and Bcl6. *PLoS One.* (2012) 7:e46126. 10.1371/journal.pone.0046126 23049957 PMC3458009

[47] Zhou J He S Wang B Yang W Zheng Y Jiang S Construction and Bioinformatics Analysis of circRNA-miRNA-mRNA Network in Acute Myocardial Infarction. *Front Genet.* (2022) 13:854993. 10.3389/fgene.2022.854993 35422846 PMC9002054

[48] Paiva S Agbulut O. MiRroring the multiple potentials of MicroRNAs in Acute Myocardial Infarction. *Front Cardiovasc Med.* (2017) 4:73. 10.3389/fcvm.2017.00073 29209617 PMC5701911

[49] Chen Z Li C Lin K Zhang Q Chen Y Rao L. MicroRNAs in acute myocardial infarction: Evident value as novel biomarkers? *Anatol J Cardiol.* (2018) 19:140–7. 10.14744/AnatolJCardiol.2017.8124 29424735 PMC5864810

[50] Wei YJ Wang JF Cheng F Xu HJ Chen JJ Xiong J miR-124-3p targeted SIRT1 to regulate cell apoptosis, inflammatory response, and oxidative stress in acute myocardial infarction in rats via modulation of the FGF21/CREB/PGC1α pathway. *J Physiol Biochem.* (2021) 77:577–87. 10.1007/s13105-021-00822-z 34146302

[51] Hu G Ma L Dong F Hu X Liu S Sun H. Inhibition of microRNA-124-3p protects against acute myocardial infarction by suppressing the apoptosis of cardiomyocytes. *Mol Med Rep.* (2019) 20:3379–87. 10.3892/mmr.2019.10565 31432169

[52] Mehdizadeh M Aguilar M Thorin E Ferbeyre G Nattel S. The role of cellular senescence in cardiac disease: basic biology and clinical relevance. *Nat Rev Cardiol.* (2022) 19:250–64. 10.1038/s41569-021-00624-2 34667279

[53] Wang Y Li L Fan LH Jing Y Li J Ouyang YC N-acetyl-L-cysteine (NAC) delays post-ovulatory oocyte aging in mouse. *Aging.* (2019) 11:2020–30. 10.18632/aging.101898 30978175 PMC6503888

[54] Wu G Li S Qu G Hua J Zong J Li X Genistein alleviates H2O2-induced senescence of human umbilical vein endothelial cells via regulating the TXNIP/NLRP3 axis. *Pharm Biol.* (2021) 59:1388–401. 10.1080/13880209.2021.1979052 34663173 PMC8526007

[55] Moreno-Gómez-Toledano R Sánchez-Esteban S Cook A Mínguez-Moratinos M Ramírez-Carracedo R Reventún P Bisphenol A Induces Accelerated Cell Aging in Murine Endothelium. *Biomolecules.* (2021) 11:1429. 10.3390/biom11101429 34680063 PMC8533150

[56] Zhang X Dong Y Li WC Tang BX Li J Zang Y. Roxithromycin attenuates bleomycin-induced pulmonary fibrosis by targeting senescent cells. *Acta Pharmacol Sin.* (2021) 42:2058–68. 10.1038/s41401-021-00618-3 33654217 PMC8633281

[57] Kurosaki Y Imoto A Kawakami F Ouchi M Morita A Yokoba M *In vitro* study on effect of bardoxolone methyl on cisplatin-induced cellular senescence in human proximal tubular cells. *Mol Cell Biochem.* (2022) 477:689–99. 10.1007/s11010-021-04295-y 34973124 PMC8857011

[58] Martin LF Richardson LS da Silva MG Sheller-Miller S Menon R. Dexamethasone induces primary amnion epithelial cell senescence through telomere-P21 associated pathway†. *Biol Reprod.* (2019) 100:1605–16. 10.1093/biolre/ioz048 30927408 PMC6561861

