# Supplementary figures and images for: Identification and validation of senescence-related genes in circulating endothelial cells of patients with acute myocardial infarction
Source: Front Cardiovasc Med. 2022 Dec 13;9:1057985. doi: 10.3389/fcvm.2022.1057985 (PMC9792765; doi:10.3389/fcvm.2022.1057985)

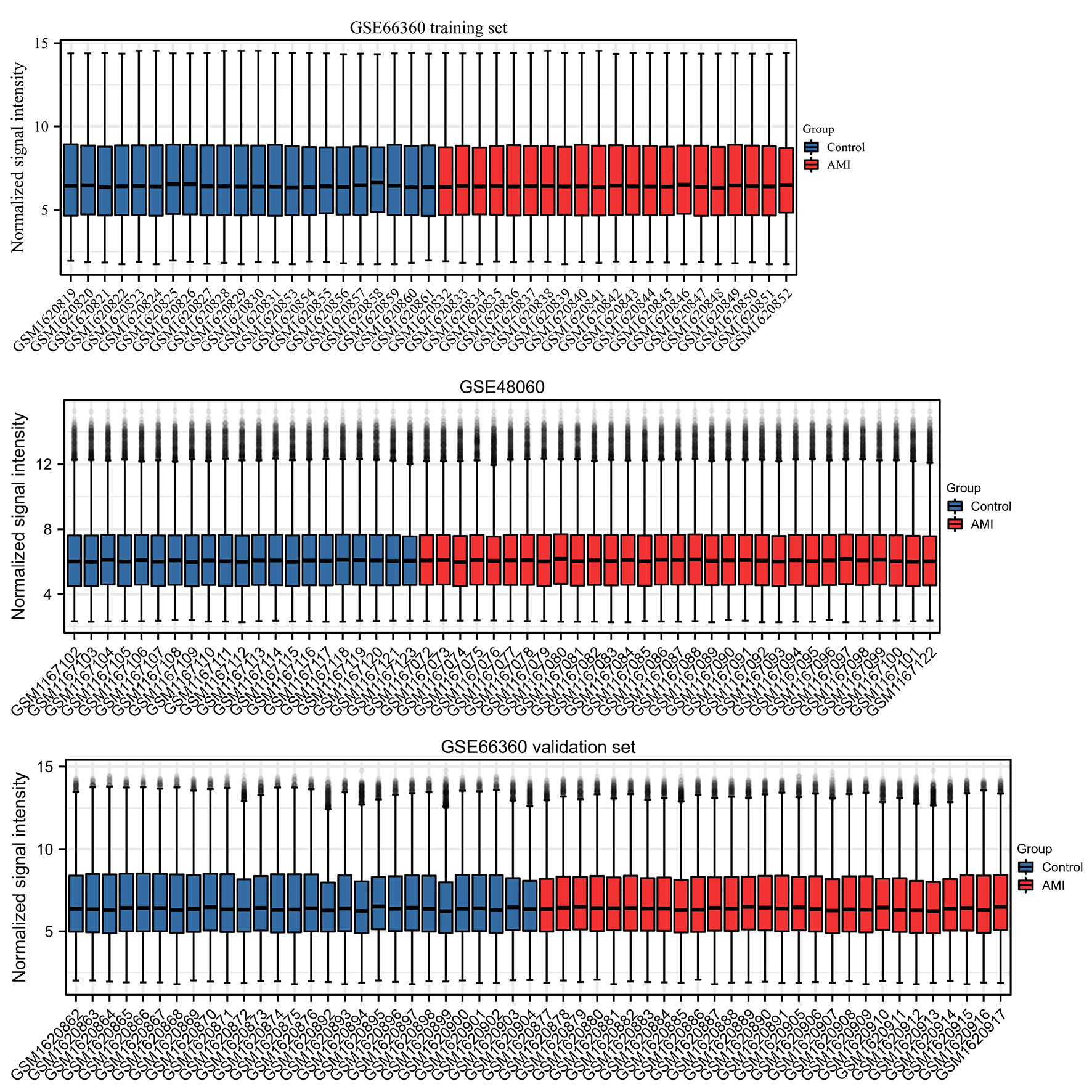

Supplement: Supplementary Figure 1 — Distribution trends for box plots of datasets. [file Image_1.TIFF]

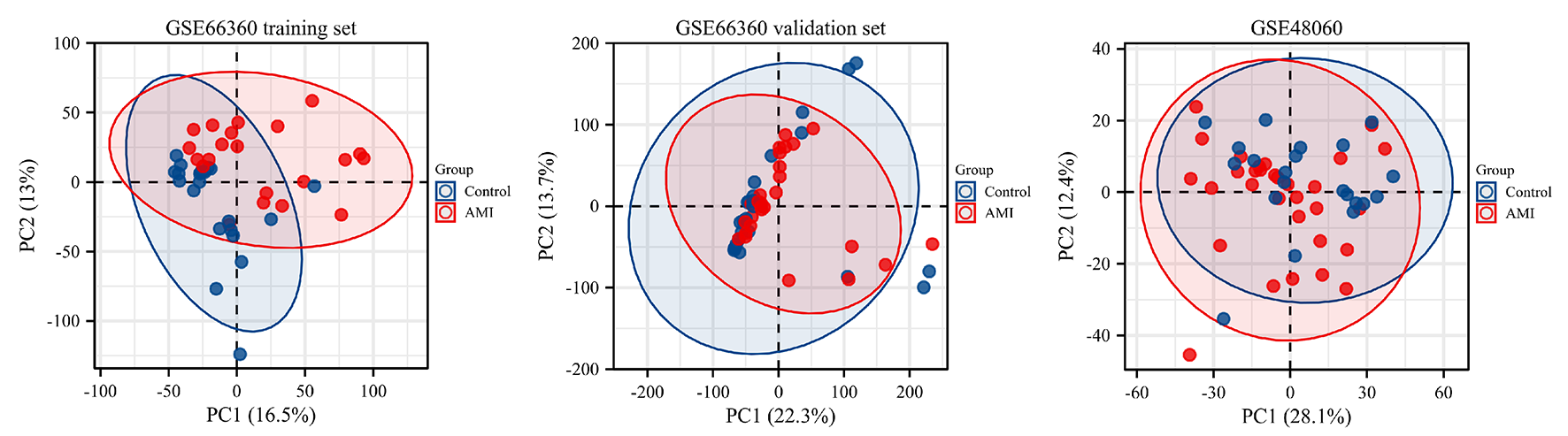

Supplement: Supplementary Figure 2 — Principal component analysis of datasets. [file Image_2.TIFF]

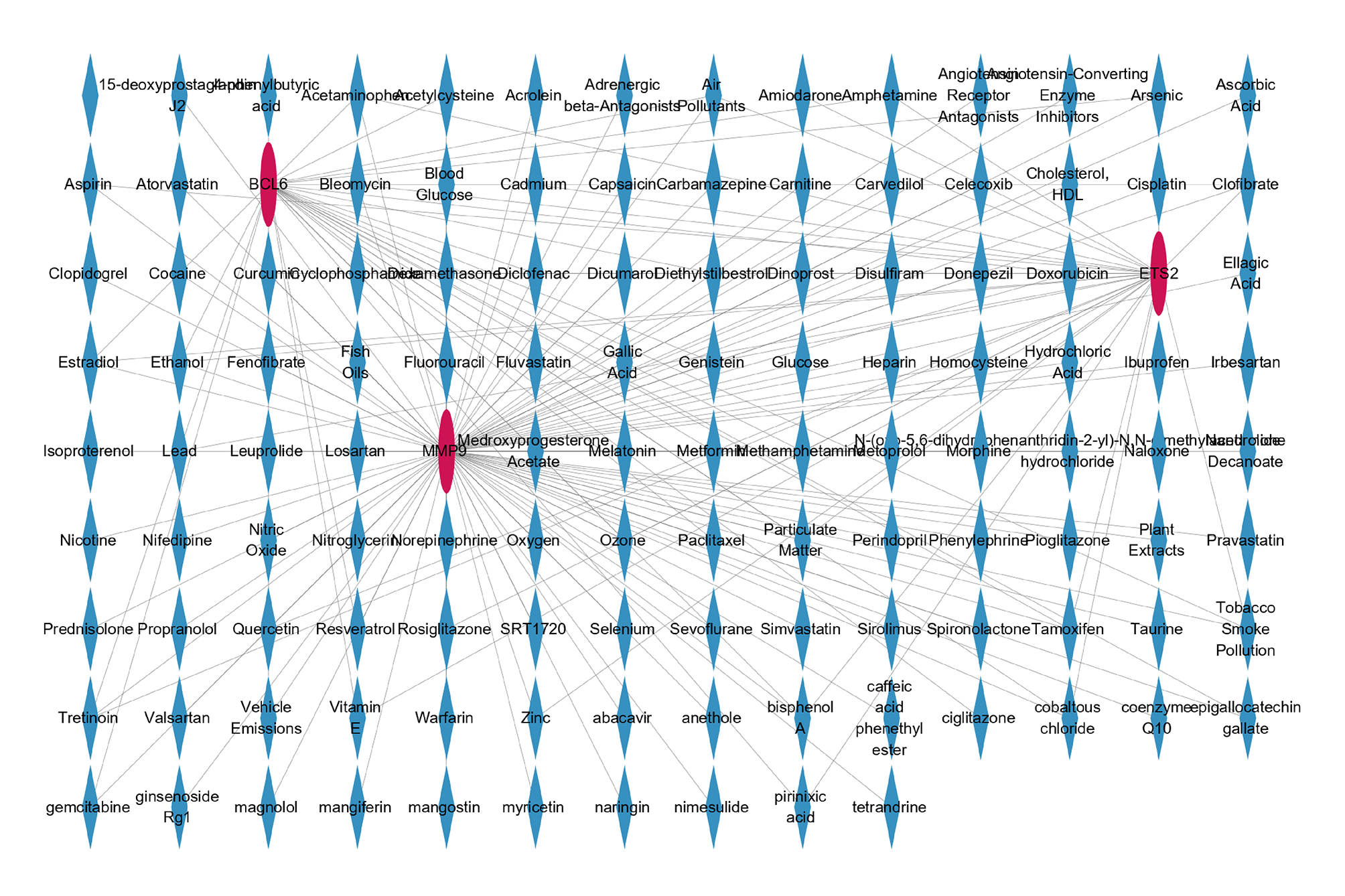

Supplement: Supplementary Figure 3 — Gene-drug interaction network constructed with three diagnostic genes and therapeutic drugs. Red ellipses represent diagnostic genes, and blue diamonds represent target drugs. [file Image_3.TIFF]

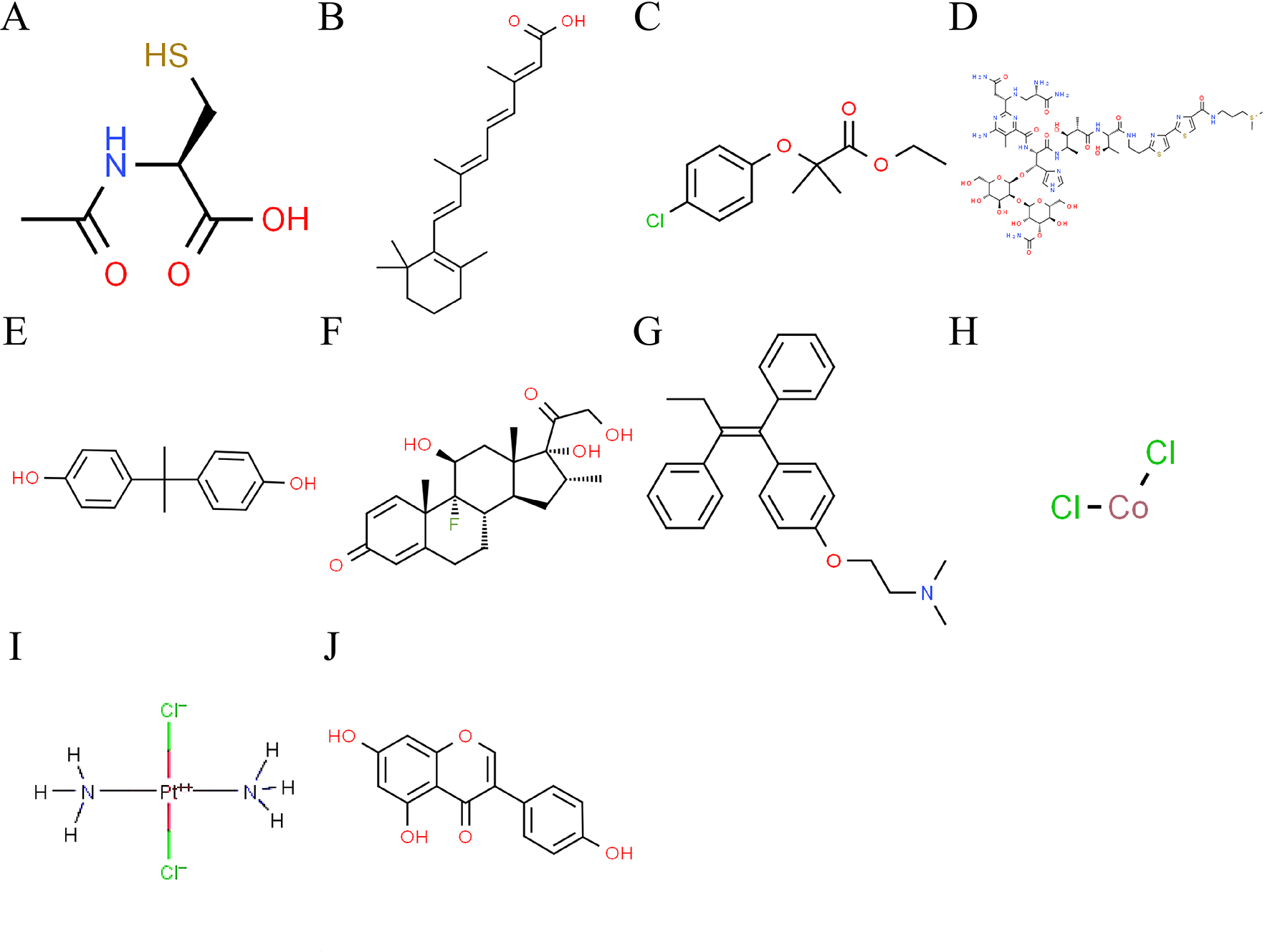

Supplement: Supplementary Figure 4 — The chemical structures of the therapeutic target drugs. (A) Acetylcysteine, (B) tretinoin, (C) clofibrate, (D) bleomycin, (E) bisphenol A, (F) dexamethasone, (G) tamoxifen, (H) cobaltous chloride, (I) cisplatin, and (J) genistein. [file Image_4.TIFF]
