# Supplementary material for: Identification and validation of senescence-related genes in circulating endothelial cells of patients with acute myocardial infarction
Source: Front Cardiovasc Med. 2022 Dec 13;9:1057985. doi: 10.3389/fcvm.2022.1057985 (PMC9792765; doi:10.3389/fcvm.2022.1057985)
Supplement: Supplementary Table 1 — Senescence-related genes. [file Table_1.DOCX]

| Gene | CSNK2A | IGFBP5 | MVK | RAD21 | TFDP1 |
| --- | --- | --- | --- | --- | --- |
| ACLY | CXCL1 | IL1A | MYC | RAF1 | TGFB1I1 |
| AAK1 | DDB2 | IL8 | MYLK | RB1 | TLR3 |
| ABI3 | CYR61 | ING1 | NADK | RBP2 | TMSB4X |
| ADCK5 | DEK | ING2 | NANOG | RBX1 | TNFSF13 |
| AKR1B1 | DGCR8 | IRF3 | NDRG1 | RNASEL | TNFSF15 |
| AGT | DHCR24 | IRF5 | NEK1 | RPS6KA6 | TOP1 |
| AKT1 | DLX2 | IRF7 | NEK4 | RSL1D1 | TP63 |
| ALOX15B | DHX9 | ITPK1 | NEK6 | RUNX1 | TPR |
| AR | DPY30 | ITGB4 | NFE2L2 | RUVBL2 | TP53 |
| ARPC1B | DUSP3 | ITPKB | NINJ1 | SENP1 | TRIM28 |
| ASF1A | DUSP16 | ITSN2 | NOTCH3 | SENP2 | TRPM8 |
| ASPH | E2F1 | KCNJ12 | NOX4 | SENP7 | TXN |
| ATF7IP | EHF | KDM4A | NR2E1 | SERPINE1 | TXNIP |
| ATM | ENDOG | KDM5B | NTN4 | SFN | UBTD1 |
| AURKA | EPHA3 | KIAA1524 | NUAK1 | SIK1 | TYK2 |
| AXL | ERRFI1 | KL | OTX2 | SGK1 | VENTX |
| BAG3 | ETS1 | KSR2 | P3H1 | SIN3B | USP1 |
| BHLHE40 | ETS2 | LATS1 | PATZ1 | SIRT1 | VEGFA |
| BCL6 | EWSR1 | LEO1 | PAK4 | SIRT6 | WNT16 |
| BLK | FASTK | LGALS3 | PBRM1 | SIX1 | WNT2 |
| BLVRA | EZH2 | LIMA1 | PCGF2 | SLC13A3 | WRN |
| BMI1 | FBXO31 | LIMK1 | PDCD10 | SLC16A7 | WT1 |
| BRAF | FOXM1 | MAGEA2 | PDIK1L | SMARCA4 | XAF1 |
| BRD7 | FOS | MAGOH | PDZD2 | SMG1 | WWP1 |
| BRCA1 | FOXO3 | MAD2L1 | PDPK1 | SMARCB1 | YAP1 |
| BTG3 | FXR1 | MAGOH | PEBP1 | SMURF2 | YPEL3 |
| C11orf31 | G6PD | MAP2K1 | PEX19 | SNAI1 | ZFP36 |
| CAV1 | GAPDH | MAP2K2 | PIAS4 | SOCS1 | ZMAT3 |
| CBX7 | GKN1 | MAP2K3 | PIK3R52A | SOD1 | ZNF148 |
| CBX8 | GATA4 | MAP2K6 | PIK3C | SORBS2 |  |
| CCND1 | GNG11 | MAP3K6 | PIM1 | SOX2 |  |
| CDK1 | GLB1 | MAP2K7 | PLA2R1 | SPIN1 |  |
| CDK18 | GRK6 | MAP4K1 | PKM | SOX5 |  |
| CDK2AP1 | HDAC4 | MAP3K7 | PML | SP1 |  |
| CDK6 | HDAC1 | MAPK12 | PNPT1 | SPOP |  |
| CDK4 | HEPACAM | MAPKAPK5 | PMVK | SRC |  |
| CDKN1A | HJURP | Mar-05 | POT1 | SREBF1 |  |
| CDKN1C | HIVEP1 | MAPK14 | POU5F1 | SRSF1 |  |
| CDKN1B | HK3 | MAST1 | PPM1B | STAT5B |  |
| CDKN2A | HMGB1 | MATK | PPM1D | STK32C |  |
| CDKN2AIP | HRAS | MCL1 | PRMT6 | STK40 |  |
| CDKN2B | HSPA5 | MDH1 | PRKCH | SUPT5H |  |
| CENPA | HSPB2 | MCRS1 | PRKCD | SYK |  |
| CEBPB | ID1 | MECP2 | PROX1 | TACC3 |  |
| CHEK1 | ID4 | MOB3A | PRPF19 | TERC |  |
| CKB | IGFBP1 | MMP9 | PSMB5 | TBX2 |  |
| CPEB1 | IFNG | MORC3 | PTRF | TERF2 |  |
| CSNK1A1 | IGFBP3 | MORF4 | PTTG1 | TERT |  |
| CTNNAL1 | IGFBP6 | MXD4 | PSMD14 | TFAP4 |  |

Supplementary table 1: 279 senescence-related genes.
