# Supplementary material for: Identification and validation of senescence-related genes in circulating endothelial cells of patients with acute myocardial infarction
Source: Front Cardiovasc Med. 2022 Dec 13;9:1057985. doi: 10.3389/fcvm.2022.1057985 (PMC9792765; doi:10.3389/fcvm.2022.1057985)
Supplement: Supplementary Table 2 — Significantly up- and downregulated genes. [file Table_2.DOCX]

| id | logFC | AveExpr | t | P.Value | adj.P.Val | B | Gene.Symbol |
| --- | --- | --- | --- | --- | --- | --- | --- |
| 212354_at | 3.673925 | 5.7992 | 5.544321 | 1.64E-06 | 0.000516 | 5.064081 | SULF1 |
| 223597_at | 3.590027 | 7.021548 | 6.267619 | 1.45E-07 | 0.000117 | 7.326407 | ITLN1 |
| 218559_s_at | 3.527331 | 8.636314 | 5.95068 | 4.22E-07 | 0.000212 | 6.333453 | MAFB |
| 225207_at | 3.395536 | 6.003238 | 6.368612 | 1.03E-07 | 0.000106 | 7.642783 | PDK4 |
| 223204_at | 3.267288 | 6.176782 | 5.618277 | 1.28E-06 | 0.000421 | 5.29451 | FAM198B |
| 204971_at | 3.202486 | 8.494374 | 4.840663 | 1.68E-05 | 0.002247 | 2.897185 | CSTA |
| 206157_at | 3.185407 | 6.359527 | 4.37778 | 7.46E-05 | 0.005854 | 1.51261 | PTX3 |
| 221731_x_at | 3.079881 | 8.872386 | 4.753832 | 2.23E-05 | 0.002682 | 2.634334 | VCAN |
| 209959_at | 3.075932 | 6.531337 | 6.518076 | 6.26E-08 | 7.53E-05 | 8.110662 | NR4A3 |
| 201842_s_at | 3.074672 | 5.002236 | 4.853208 | 1.61E-05 | 0.002211 | 2.935263 | EFEMP1 |
| 202291_s_at | 3.034624 | 8.1058 | 5.170577 | 5.69E-06 | 0.001141 | 3.906188 | MGP |
| 205863_at | 3.002281 | 8.269098 | 5.724962 | 8.99E-07 | 0.000349 | 5.627456 | S100A12 |
| 201909_at | 2.945483 | 9.541962 | 2.664888 | 0.010779 | 0.118941 | -3.01537 | RPS4Y1 |
| 205000_at | 2.92177 | 8.643489 | 2.809399 | 0.007421 | 0.095392 | -2.68522 | DDX3Y |
| 205403_at | 2.84137 | 8.422821 | 6.606394 | 4.65E-08 | 7.17E-05 | 8.386843 | IL1R2 |
| 235086_at | 2.835569 | 6.592245 | 5.316176 | 3.51E-06 | 0.000836 | 4.355746 | THBS1 |
| 201667_at | 2.810262 | 6.48389 | 5.090393 | 7.41E-06 | 0.001372 | 3.659618 | GJA1 |
| 205568_at | 2.729441 | 8.229873 | 5.794167 | 7.13E-07 | 0.000286 | 5.843726 | AQP9 |
| 206700_s_at | 2.696536 | 7.819397 | 2.364573 | 0.022604 | 0.180023 | -3.66098 | KDM5D |
| 220034_at | 2.683776 | 6.74177 | 6.181018 | 1.94E-07 | 0.00014 | 7.055048 | IRAK3 |
| 209774_x_at | 2.666382 | 8.800521 | 4.693341 | 2.71E-05 | 0.003141 | 2.451997 | CXCL2 |
| 217591_at | 2.651767 | 9.162966 | 7.984866 | 4.75E-10 | 3.43E-06 | 12.63326 | SKIL |
| 205476_at | 2.648274 | 8.270055 | 5.184502 | 5.43E-06 | 0.001121 | 3.949085 | CCL20 |
| 210164_at | 2.64633 | 8.178742 | 5.132923 | 6.44E-06 | 0.001257 | 3.790305 | GZMB |
| 204409_s_at | 2.637609 | 7.031134 | 2.826103 | 0.007103 | 0.09256 | -2.64629 | EIF1AY |
| 202912_at | 2.631842 | 7.842079 | 5.937665 | 4.40E-07 | 0.000216 | 6.292703 | ADM |
| 222934_s_at | 2.622517 | 9.894725 | 6.335259 | 1.16E-07 | 0.000106 | 7.538316 | CLEC4E |
| 203887_s_at | 2.618021 | 6.640103 | 6.53277 | 5.96E-08 | 7.53E-05 | 8.156629 | THBD |
| 219434_at | 2.570718 | 7.770022 | 5.60151 | 1.36E-06 | 0.000432 | 5.242236 | TREM1 |
| 228492_at | 2.555259 | 7.1176 | 2.46291 | 0.017834 | 0.157152 | -3.45585 | USP9Y |
| 204232_at | 2.540294 | 9.889629 | 5.847683 | 5.96E-07 | 0.000253 | 6.011092 | FCER1G |
| 1552773_at | 2.537271 | 6.244488 | 5.042702 | 8.67E-06 | 0.001503 | 3.513342 | CLEC4D |
| 209684_at | 2.523102 | 5.844543 | 5.611752 | 1.31E-06 | 0.000424 | 5.274164 | RIN2 |
| 206522_at | 2.443384 | 8.269408 | 5.678061 | 1.05E-06 | 0.000377 | 5.481013 | MGAM |
| 223454_at | 2.427009 | 8.717273 | 8.384882 | 1.29E-10 | 1.75E-06 | 13.83132 | CXCL16 |
| 210845_s_at | 2.426581 | 7.983886 | 5.988306 | 3.72E-07 | 0.000199 | 6.451278 | PLAUR |
| 211429_s_at | 2.399225 | 8.81942 | 4.626778 | 3.36E-05 | 0.003588 | 2.25214 | SERPINA1 |
| 215049_x_at | 2.368683 | 6.332766 | 4.564624 | 4.11E-05 | 0.003972 | 2.066304 | CD163 |
| 206111_at | 2.368524 | 6.57472 | 4.085899 | 0.000187 | 0.010227 | 0.664728 | RNASE2 |
| 206026_s_at | 2.357165 | 6.226795 | 4.387185 | 7.24E-05 | 0.005759 | 1.540292 | TNFAIP6 |
| 202436_s_at | 2.353792 | 6.849121 | 5.840187 | 6.11E-07 | 0.000254 | 5.987641 | CYP1B1 |
| 202112_at | 2.344445 | 6.49437 | 4.892292 | 1.42E-05 | 0.002064 | 3.054056 | VWF |
| 202990_at | 2.343896 | 7.532215 | 5.394632 | 2.71E-06 | 0.000698 | 4.598851 | PYGL |
| 200878_at | 2.337292 | 6.677799 | 6.421084 | 8.67E-08 | 9.88E-05 | 7.807097 | EPAS1 |
| 218454_at | 2.325175 | 8.601186 | 4.500386 | 5.05E-05 | 0.004498 | 1.875073 | PLBD1 |
| 236213_at | 2.320097 | 6.398108 | 5.429393 | 2.41E-06 | 0.00065 | 4.706735 | AC079305.10 |
| 205174_s_at | 2.281968 | 7.124667 | 4.678236 | 2.85E-05 | 0.003213 | 2.406571 | QPCT |
| 204351_at | 2.276312 | 8.67176 | 4.888535 | 1.44E-05 | 0.002076 | 3.042627 | S100P |
| 209369_at | 2.274077 | 5.691507 | 5.013829 | 9.54E-06 | 0.001622 | 3.42493 | ANXA3 |
| 201743_at | 2.265243 | 7.347966 | 4.535075 | 4.52E-05 | 0.004253 | 1.978232 | CD14 |
| 1553297_a_at | 2.26502 | 8.004281 | 4.859996 | 1.58E-05 | 0.002191 | 2.955877 | CSF3R |
| 220528_at | 2.252248 | 7.110202 | 5.79357 | 7.14E-07 | 0.000286 | 5.841857 | VNN3 |
| 205237_at | 2.235669 | 7.596516 | 4.419647 | 6.53E-05 | 0.005358 | 1.635998 | FCN1 |
| 222722_at | 2.222362 | 4.381298 | 4.474876 | 5.48E-05 | 0.004744 | 1.799379 | OGN |
| 205119_s_at | 2.206124 | 9.179427 | 4.871373 | 1.52E-05 | 0.002152 | 2.990444 | FPR1 |
| 210809_s_at | 2.204646 | 5.133297 | 3.089849 | 0.003493 | 0.061119 | -2.01167 | POSTN |
| 208146_s_at | 2.1981 | 6.142405 | 3.839774 | 0.000397 | 0.01655 | -0.03113 | CPVL |
| 204622_x_at | 2.197996 | 8.487274 | 5.369396 | 2.94E-06 | 0.000737 | 4.520596 | NR4A2 |
| 203936_s_at | 2.192868 | 6.874238 | 6.803402 | 2.40E-08 | 4.73E-05 | 9.00183 | MMP9 |
| 221841_s_at | 2.188776 | 6.945891 | 4.286181 | 9.97E-05 | 0.006943 | 1.244133 | KLF4 |
| 212224_at | 2.182364 | 5.601707 | 4.42244 | 6.47E-05 | 0.005348 | 1.644243 | ALDH1A1 |
| 205239_at | 2.171748 | 6.121665 | 5.932458 | 4.48E-07 | 0.000216 | 6.276401 | AREG |
| 39402_at | 2.171604 | 9.82809 | 4.247536 | 0.000113 | 0.007619 | 1.131496 | IL1B |
| 201425_at | 2.165392 | 7.30917 | 4.765398 | 2.15E-05 | 0.002653 | 2.669273 | ALDH2 |
| 244840_x_at | 2.165045 | 7.594516 | 7.164346 | 7.18E-09 | 1.81E-05 | 10.12317 | DOCK4 |
| 219892_at | 2.159295 | 5.681378 | 4.598441 | 3.68E-05 | 0.003755 | 2.167319 | TM6SF1 |
| 216442_x_at | 2.15822 | 6.855425 | 4.758623 | 2.20E-05 | 0.002671 | 2.648804 | FN1 |
| 1558549_s_at | 2.156365 | 5.529927 | 5.050198 | 8.46E-06 | 0.00149 | 3.536316 | VNN1 |
| 203574_at | 2.149024 | 9.277555 | 7.78799 | 9.07E-10 | 4.91E-06 | 12.03695 | NFIL3 |
| 220088_at | 2.120369 | 9.912824 | 4.549253 | 4.32E-05 | 0.004136 | 2.020468 | C5AR1 |
| 201785_at | 2.119189 | 6.455114 | 5.074745 | 7.81E-06 | 0.001409 | 3.611592 | RNASE1 |
| 224724_at | 2.115357 | 6.626759 | 4.100849 | 0.000178 | 0.009914 | 0.707594 | SULF2 |
| 221060_s_at | 2.103161 | 7.349265 | 4.937667 | 1.22E-05 | 0.001907 | 3.192269 | TLR4 |
| 207850_at | 2.093941 | 6.132324 | 4.666576 | 2.96E-05 | 0.003251 | 2.371535 | CXCL3 |
| 38037_at | 2.093699 | 6.694792 | 7.361526 | 3.72E-09 | 1.34E-05 | 10.73193 | HBEGF |
| 203535_at | 2.088019 | 9.900113 | 4.914089 | 1.32E-05 | 0.001981 | 3.120413 | S100A9 |
| 229934_at | 2.086096 | 7.469714 | 4.020261 | 0.000229 | 0.011744 | 0.477311 | mir-223 |
| 226420_at | 2.070847 | 5.32708 | 4.428819 | 6.34E-05 | 0.005267 | 1.663083 | MECOM |
| 207857_at | 2.067496 | 7.035034 | 4.037118 | 0.000217 | 0.011398 | 0.52532 | LILRA2 |
| 207610_s_at | 2.048284 | 5.757241 | 3.825321 | 0.000415 | 0.016891 | -0.07138 | EMR2 |
| 224356_x_at | 2.038653 | 8.731331 | 3.89718 | 0.000333 | 0.014734 | 0.129445 | MS4A6A |
| 206371_at | 2.037129 | 6.149324 | 4.357378 | 7.96E-05 | 0.006091 | 1.452634 | FOLR3 |
| 209386_at | 2.036634 | 6.963778 | 4.109911 | 0.000173 | 0.009714 | 0.733609 | TM4SF1 |
| 227889_at | 2.031631 | 5.49423 | 3.788683 | 0.000463 | 0.018209 | -0.1731 | LPCAT2 |
| 205896_at | 2.028947 | 5.81443 | 4.126394 | 0.000164 | 0.009448 | 0.780986 | SLC22A4 |
| 212681_at | 2.013384 | 5.178471 | 4.667613 | 2.95E-05 | 0.003251 | 2.374647 | EPB41L3 |
| 235072_s_at | 1.990134 | 7.611792 | 3.624487 | 0.000757 | 0.024428 | -0.62305 | KIF13A |
| 221541_at | 1.967505 | 8.029004 | 4.19739 | 0.000132 | 0.008285 | 0.985919 | CRISPLD2 |
| 208792_s_at | 1.958974 | 9.47184 | 4.863271 | 1.56E-05 | 0.002181 | 2.965827 | CLU |
| 213095_x_at | 1.958053 | 9.319072 | 4.275898 | 0.000103 | 0.007125 | 1.214122 | AIF1 |
| 206765_at | 1.953276 | 8.203373 | 3.799573 | 0.000448 | 0.017782 | -0.14291 | KCNJ2 |
| 204614_at | 1.951382 | 4.217541 | 3.472179 | 0.001183 | 0.031453 | -1.03121 | SERPINB2 |
| 202917_s_at | 1.946266 | 11.83946 | 5.462095 | 2.16E-06 | 0.000633 | 4.808313 | S100A8 |
| 230206_at | 1.935635 | 8.238291 | 4.360389 | 7.88E-05 | 0.006076 | 1.461479 | DOCK5 |
| 205220_at | 1.931835 | 8.26594 | 3.630649 | 0.000743 | 0.024143 | -0.60634 | HCAR3 |
| 200795_at | 1.930847 | 5.572674 | 3.56483 | 0.000903 | 0.027376 | -0.78402 | SPARCL1 |
| 222218_s_at | 1.925227 | 8.133705 | 4.551536 | 4.28E-05 | 0.004124 | 2.027272 | PILRA |
| 239085_at | 1.924617 | 5.984488 | 5.347841 | 3.16E-06 | 0.000761 | 4.453798 | JDP2 |
| 205627_at | 1.919294 | 6.694845 | 4.949848 | 1.18E-05 | 0.001887 | 3.229422 | CDA |
| 238066_at | 1.916127 | 7.93545 | 4.601392 | 3.65E-05 | 0.003746 | 2.176144 | RBP7 |
| 201631_s_at | 1.91207 | 11.28948 | 4.690045 | 2.74E-05 | 0.003158 | 2.442081 | IER3 |
| 209189_at | 1.911577 | 9.346792 | 4.587576 | 3.82E-05 | 0.003839 | 2.134839 | FOS |
| 223484_at | 1.904066 | 7.494541 | 4.035335 | 0.000218 | 0.011398 | 0.520239 | C15orf48 |
| 213975_s_at | 1.903706 | 10.92509 | 4.198137 | 0.000131 | 0.008285 | 0.988083 | LYZ |
| 202878_s_at | 1.900845 | 8.177882 | 4.119694 | 0.000168 | 0.009571 | 0.761716 | CD93 |
| 204103_at | 1.900434 | 9.458246 | 4.618922 | 3.45E-05 | 0.003638 | 2.228608 | CCL4 |
| 229967_at | 1.889573 | 7.961731 | 4.327856 | 8.74E-05 | 0.006351 | 1.366027 | CMTM2 |
| 1553043_a_at | 1.881467 | 6.039024 | 3.581375 | 0.00086 | 0.02637 | -0.73951 | CD300LF |
| 36711_at | 1.881079 | 8.847736 | 4.823549 | 1.78E-05 | 0.002347 | 2.845277 | MAFF |
| 209099_x_at | 1.877935 | 7.79637 | 5.67489 | 1.06E-06 | 0.000377 | 5.471116 | JAG1 |
| 220005_at | 1.870912 | 5.346715 | 3.359944 | 0.001636 | 0.03822 | -1.32586 | P2RY13 |
| 212657_s_at | 1.861395 | 10.55414 | 4.771798 | 2.10E-05 | 0.002647 | 2.688616 | IL1RN |
| 1569095_at | 1.85811 | 6.486428 | 4.36779 | 7.70E-05 | 0.005978 | 1.483231 | LOC731424 |
| 207075_at | 1.857286 | 8.158753 | 4.946438 | 1.19E-05 | 0.001894 | 3.21902 | NLRP3 |
| 203139_at | 1.853349 | 7.474714 | 3.806565 | 0.000439 | 0.017539 | -0.12351 | DAPK1 |
| 200665_s_at | 1.851485 | 9.064407 | 4.076563 | 0.000192 | 0.010396 | 0.637994 | SPARC |
| 204924_at | 1.851125 | 7.683225 | 4.654605 | 3.07E-05 | 0.003363 | 2.335588 | TLR2 |
| 218718_at | 1.850774 | 5.228361 | 3.574194 | 0.000878 | 0.026761 | -0.75884 | PDGFC |
| 202637_s_at | 1.8481 | 8.097312 | 5.125781 | 6.60E-06 | 0.001262 | 3.768342 | ICAM1 |
| 205715_at | 1.83437 | 6.863342 | 4.027445 | 0.000224 | 0.011526 | 0.497761 | BST1 |
| 209396_s_at | 1.832675 | 7.18261 | 3.898106 | 0.000332 | 0.014723 | 0.132044 | CHI3L1 |
| 210772_at | 1.824492 | 5.742244 | 3.492746 | 0.001115 | 0.030479 | -0.97664 | FPR2 |
| 228648_at | 1.813406 | 6.093378 | 6.084663 | 2.69E-07 | 0.000171 | 6.753125 | LRG1 |
| 206715_at | 1.812086 | 6.836663 | 3.58481 | 0.000851 | 0.026253 | -0.73026 | TFEC |
| 205681_at | 1.810085 | 10.95849 | 5.632349 | 1.23E-06 | 0.000412 | 5.33839 | BCL2A1 |
| 203435_s_at | 1.806142 | 7.465503 | 4.787718 | 2.00E-05 | 0.002574 | 2.736759 | MME |
| 241889_at | 1.803316 | 6.760548 | 5.174393 | 5.62E-06 | 0.001141 | 3.917942 | NFKBID |
| 236399_at | 1.80218 | 4.858956 | 4.610606 | 3.54E-05 | 0.003698 | 2.203713 | RP11-443B7.1 |
| 229770_at | 1.799391 | 6.549895 | 4.233281 | 0.000118 | 0.007894 | 1.090046 | GLT1D1 |
| 206932_at | 1.796917 | 4.581917 | 5.105614 | 7.05E-06 | 0.001328 | 3.706365 | CH25H |
| 201963_at | 1.793552 | 8.681216 | 5.513297 | 1.82E-06 | 0.000564 | 4.967522 | ACSL1 |
| 211816_x_at | 1.791633 | 7.290604 | 4.522832 | 4.70E-05 | 0.004404 | 1.941793 | FCAR |
| 214131_at | 1.784411 | 7.415209 | 2.093147 | 0.042231 | 0.251373 | -4.19332 | TXLNGY |
| 202510_s_at | 1.781192 | 9.114093 | 3.777978 | 0.000479 | 0.018621 | -0.20273 | TNFAIP2 |
| 202381_at | 1.777433 | 6.661684 | 5.439558 | 2.33E-06 | 0.000639 | 4.7383 | ADAM9 |
| 202284_s_at | 1.766373 | 7.973972 | 5.649483 | 1.16E-06 | 0.000398 | 5.391837 | CDKN1A |
| 206515_at | 1.764236 | 6.182341 | 3.759463 | 0.000506 | 0.019289 | -0.25388 | CYP4F3 |
| 204122_at | 1.756217 | 10.29327 | 4.24078 | 0.000115 | 0.007735 | 1.111843 | TYROBP |
| 220945_x_at | 1.756108 | 7.3671 | 3.815081 | 0.000428 | 0.017188 | -0.09985 | MANSC1 |
| 225283_at | 1.743854 | 5.65349 | 4.059985 | 0.000202 | 0.010863 | 0.590584 | ARRDC4 |
| 218660_at | 1.743691 | 7.539733 | 4.382353 | 7.35E-05 | 0.005791 | 1.526067 | DYSF |
| 220001_at | 1.729842 | 6.330308 | 4.160752 | 0.000148 | 0.008744 | 0.879989 | PADI4 |
| 212598_at | 1.727662 | 5.962153 | 4.911055 | 1.34E-05 | 0.001981 | 3.111169 | WDFY3 |
| 229560_at | 1.714306 | 6.459116 | 3.416588 | 0.00139 | 0.0348 | -1.17782 | TLR8 |
| 230170_at | 1.707506 | 8.238192 | 4.918664 | 1.30E-05 | 0.001981 | 3.134349 | OSM |
| 211434_s_at | 1.698864 | 7.029641 | 4.957472 | 1.15E-05 | 0.001868 | 3.252688 | CCRL2 |
| 203561_at | 1.69603 | 10.62599 | 4.705002 | 2.61E-05 | 0.003041 | 2.487096 | FCGR2A |
| 201162_at | 1.68708 | 6.824101 | 4.57754 | 3.94E-05 | 0.003839 | 2.104858 | IGFBP7 |
| 224818_at | 1.684571 | 6.420895 | 4.597707 | 3.69E-05 | 0.003755 | 2.165124 | SORT1 |
| 226841_at | 1.679879 | 8.653435 | 3.345147 | 0.001707 | 0.039321 | -1.3643 | MPEG1 |
| 220384_at | 1.677327 | 5.463904 | 3.923767 | 0.000307 | 0.013926 | 0.20418 | NME8 |
| 209791_at | 1.673273 | 8.32716 | 3.686538 | 0.000629 | 0.021949 | -0.45417 | PADI2 |
| 227235_at | 1.672543 | 5.337349 | 4.471901 | 5.53E-05 | 0.00477 | 1.79056 | GUCY1A3 |
| 207630_s_at | 1.664087 | 9.141986 | 5.265849 | 4.15E-06 | 0.000936 | 4.200106 | CREM |
| 220404_at | 1.66267 | 6.774426 | 5.304253 | 3.65E-06 | 0.00086 | 4.318853 | GPR97 |
| 217521_at | 1.66183 | 7.411275 | 4.961288 | 1.13E-05 | 0.001859 | 3.264338 | HAL |
| 209101_at | 1.657113 | 5.428079 | 4.497896 | 5.09E-05 | 0.004516 | 1.867678 | CTGF |
| 243099_at | 1.655458 | 6.889092 | 4.206251 | 0.000128 | 0.008285 | 1.011596 | NFAM1 |
| 230760_at | 1.653654 | 5.794698 | 2.407597 | 0.020391 | 0.168885 | -3.57201 | ZFY |
| 221958_s_at | 1.651903 | 5.652998 | 3.548422 | 0.000947 | 0.028409 | -0.82805 | WLS |
| 221698_s_at | 1.651842 | 9.001988 | 3.455332 | 0.001243 | 0.032387 | -1.07578 | CLEC7A |
| 228153_at | 1.650713 | 9.606718 | 4.941186 | 1.21E-05 | 0.001907 | 3.203 | RNF144B |
| 226237_at | 1.64951 | 3.798801 | 4.175781 | 0.000141 | 0.008561 | 0.923397 | COL8A1 |
| 218995_s_at | 1.644134 | 7.040619 | 5.991562 | 3.67E-07 | 0.000199 | 6.461476 | EDN1 |
| 206934_at | 1.643701 | 7.412063 | 4.170495 | 0.000143 | 0.008561 | 0.908121 | SIRPB1 |
| 225955_at | 1.640159 | 7.066122 | 6.726738 | 3.11E-08 | 5.61E-05 | 8.762715 | METRNL |
| 206120_at | 1.639885 | 5.703678 | 3.682495 | 0.000637 | 0.022073 | -0.46522 | CD33 |
| 201360_at | 1.6398 | 7.426531 | 4.295633 | 9.68E-05 | 0.006826 | 1.271739 | CST3 |
| 206733_at | 1.633322 | 6.532993 | 3.867941 | 0.000364 | 0.015678 | 0.047522 | TULP2 |
| 213032_at | 1.631108 | 5.966537 | 3.25524 | 0.002203 | 0.045923 | -1.59576 | NFIB |
| 202888_s_at | 1.630128 | 6.767243 | 4.634253 | 3.28E-05 | 0.003537 | 2.274541 | ANPEP |
| 204470_at | 1.628077 | 8.205374 | 2.713412 | 0.009521 | 0.110725 | -2.90586 | CXCL1 |
| 213355_at | 1.62621 | 5.563175 | 3.137136 | 0.003066 | 0.055885 | -1.89411 | ST3GAL6 |
| 1554503_a_at | 1.624912 | 5.406213 | 4.421423 | 6.49E-05 | 0.005348 | 1.641239 | OSCAR |
| 204214_s_at | 1.624544 | 5.810088 | 4.353434 | 8.06E-05 | 0.006125 | 1.441051 | RAB32 |
| 208092_s_at | 1.616459 | 7.442488 | 4.189493 | 0.000135 | 0.008358 | 0.963055 | FAM49A |
| 203765_at | 1.616073 | 8.035052 | 4.34561 | 8.26E-05 | 0.006213 | 1.418086 | GCA |
| 222258_s_at | 1.614086 | 3.592132 | 4.576619 | 3.95E-05 | 0.003839 | 2.102107 | SH3BP4 |
| 230559_x_at | 1.610676 | 6.123994 | 4.196522 | 0.000132 | 0.008285 | 0.983405 | FGD4 |
| 235568_at | 1.61002 | 7.101995 | 4.5769 | 3.95E-05 | 0.003839 | 2.102947 | MCEMP1 |
| 208131_s_at | 1.607028 | 7.062395 | 2.874093 | 0.006258 | 0.085569 | -2.53359 | PTGIS |
| 231579_s_at | 1.604551 | 9.50034 | 4.337706 | 8.47E-05 | 0.006304 | 1.394899 | TIMP2 |
| 227099_s_at | 1.602316 | 5.730735 | 4.672194 | 2.90E-05 | 0.003242 | 2.388411 | C11orf96 |
| 223836_at | 1.595392 | 7.360567 | 3.053381 | 0.003861 | 0.064564 | -2.10158 | FGFBP2 |
| 204748_at | 1.592604 | 9.929816 | 2.991499 | 0.004569 | 0.070503 | -2.25259 | PTGS2 |
| 206115_at | 1.592286 | 7.743995 | 3.032281 | 0.00409 | 0.067198 | -2.15329 | EGR3 |
| 204588_s_at | 1.585592 | 8.206219 | 4.255914 | 0.00011 | 0.007463 | 1.155883 | SLC7A7 |
| 209949_at | 1.577163 | 9.222284 | 4.015159 | 0.000232 | 0.01183 | 0.462797 | NCF2 |
| 37028_at | 1.575875 | 9.339533 | 4.867939 | 1.54E-05 | 0.002162 | 2.980009 | PPP1R15A |
| 204440_at | 1.570728 | 9.835145 | 5.258019 | 4.26E-06 | 0.000951 | 4.175916 | CD83 |
| 201341_at | 1.569103 | 6.249823 | 3.672105 | 0.000657 | 0.022457 | -0.49358 | ENC1 |
| 210222_s_at | 1.566836 | 5.359647 | 3.88933 | 0.000341 | 0.015029 | 0.107424 | RTN1 |
| 214146_s_at | 1.566522 | 10.62683 | 3.465792 | 0.001206 | 0.031723 | -1.04812 | PPBP |
| 209160_at | 1.562044 | 7.054496 | 4.846132 | 1.65E-05 | 0.002235 | 2.913782 | AKR1C3 |
| 227265_at | 1.561699 | 8.937638 | 3.863548 | 0.000369 | 0.015775 | 0.035238 | FGL2 |
| 209383_at | 1.561298 | 8.577798 | 5.083299 | 7.59E-06 | 0.001391 | 3.637842 | DDIT3 |
| 206214_at | 1.555741 | 4.553031 | 3.476687 | 0.001168 | 0.031249 | -1.01926 | PLA2G7 |
| 202897_at | 1.550522 | 7.563834 | 4.442228 | 6.08E-05 | 0.005162 | 1.702714 | SIRPA |
| 204141_at | 1.547056 | 7.475307 | 3.66537 | 0.00067 | 0.022862 | -0.51194 | TUBB2A |
| 212501_at | 1.545117 | 9.871299 | 5.888679 | 5.19E-07 | 0.000234 | 6.139365 | CEBPB |
| 207697_x_at | 1.543532 | 8.320954 | 4.647931 | 3.14E-05 | 0.003419 | 2.31556 | LILRB2 |
| 203305_at | 1.541536 | 7.146488 | 2.897478 | 0.005881 | 0.082748 | -2.47821 | F13A1 |
| 31874_at | 1.540626 | 6.92952 | 4.043128 | 0.000213 | 0.011332 | 0.542458 | GAS2L1 |
| 204007_at | 1.540425 | 9.844436 | 3.637118 | 0.000729 | 0.02388 | -0.58879 | FCGR3B |
| 236193_at | 1.54 | 5.586571 | 3.500432 | 0.00109 | 0.030121 | -0.9562 | HIST1H2BC |
| 232829_at | 1.536555 | 3.922456 | 3.427214 | 0.001348 | 0.034107 | -1.1499 | OR52K3P |
| 210660_at | 1.53608 | 6.889799 | 3.778711 | 0.000477 | 0.018621 | -0.2007 | LILRA1 |
| 203504_s_at | 1.530198 | 6.612186 | 4.631266 | 3.32E-05 | 0.003554 | 2.265589 | ABCA1 |
| 205479_s_at | 1.528706 | 5.935964 | 3.520882 | 0.001027 | 0.029412 | -0.9017 | PLAU |
| 208891_at | 1.524038 | 8.512559 | 3.008918 | 0.004358 | 0.069252 | -2.21028 | DUSP6 |
| 1564052_at | 1.5239 | 7.15004 | 2.996156 | 0.004512 | 0.07002 | -2.2413 | TREML4 |
| 201531_at | 1.520505 | 10.82945 | 7.690918 | 1.25E-09 | 5.41E-06 | 11.74143 | ZFP36 |
| 1569583_at | 1.518814 | 6.736146 | 4.911325 | 1.33E-05 | 0.001981 | 3.111993 | EREG |
| 227276_at | 1.517922 | 7.216577 | 3.832075 | 0.000406 | 0.016666 | -0.05258 | PLXDC2 |
| 221802_s_at | 1.511362 | 5.95923 | 4.12297 | 0.000166 | 0.009512 | 0.771138 | KIAA1598 |
| 201324_at | 1.511324 | 6.396636 | 3.604267 | 0.000804 | 0.025366 | -0.67776 | EMP1 |
| 204081_at | 1.511181 | 8.145848 | 3.593344 | 0.00083 | 0.025896 | -0.70725 | NRGN |
| 203021_at | 1.505302 | 6.23698 | 4.50266 | 5.01E-05 | 0.004498 | 1.881827 | SLPI |
| 208782_at | 1.501883 | 5.635576 | 3.73442 | 0.000545 | 0.020088 | -0.32288 | FSTL1 |
| 1552398_a_at | 1.498554 | 6.828953 | 2.492121 | 0.016604 | 0.150457 | -3.39371 | CLEC12A |
| 238439_at | 1.497034 | 5.569675 | 4.885202 | 1.45E-05 | 0.002084 | 3.03249 | ANKRD22 |
| 202768_at | 1.496786 | 8.061415 | 3.968329 | 0.000268 | 0.013049 | 0.329953 | FOSB |
| 243296_at | 1.496334 | 11.69648 | 5.012298 | 9.58E-06 | 0.001622 | 3.420244 | NAMPT |
| 1569827_at | 1.491043 | 7.733786 | 3.478263 | 0.001163 | 0.031249 | -1.01508 | ATG7 |
| 229584_at | 1.490383 | 9.00973 | 3.013689 | 0.004302 | 0.068871 | -2.19867 | LRRK2 |
| 224836_at | 1.485113 | 8.401844 | 4.386372 | 7.26E-05 | 0.005759 | 1.537899 | TP53INP2 |
| 226397_s_at | 1.483025 | 10.26327 | 4.285098 | 0.0001 | 0.006943 | 1.240972 | PHACTR1 |
| 219761_at | 1.480317 | 3.513379 | 3.394145 | 0.001483 | 0.036046 | -1.23664 | CLEC1A |
| 213560_at | 1.479009 | 9.57459 | 5.281935 | 3.94E-06 | 0.000897 | 4.249826 | GADD45B |
| 233217_at | 1.478734 | 7.712337 | 3.279108 | 0.00206 | 0.043986 | -1.53467 | CTC-510F12.4 |
| 212188_at | 1.477595 | 8.498246 | 3.252903 | 0.002218 | 0.046094 | -1.60172 | KCTD12 |
| 207008_at | 1.474814 | 7.826434 | 3.278678 | 0.002062 | 0.043996 | -1.53577 | CXCR2 |
| 204959_at | 1.473617 | 8.526855 | 2.740243 | 0.008884 | 0.106529 | -2.84472 | MNDA |
| 203140_at | 1.471151 | 10.10158 | 5.504258 | 1.88E-06 | 0.000573 | 4.939402 | BCL6 |
| 226489_at | 1.463753 | 6.958331 | 5.651978 | 1.15E-06 | 0.000398 | 5.399619 | TMCC3 |
| 227184_at | 1.461138 | 7.908418 | 3.425746 | 0.001354 | 0.034161 | -1.15376 | PTAFR |
| 226322_at | 1.460957 | 3.903779 | 3.98566 | 0.000254 | 0.012542 | 0.379038 | TMTC1 |
| 1557049_at | 1.457894 | 5.738272 | 4.255174 | 0.00011 | 0.007463 | 1.153727 | BTBD19 |
| 206380_s_at | 1.456195 | 7.549231 | 3.845322 | 0.00039 | 0.016351 | -0.01566 | CFP |
| 208438_s_at | 1.455291 | 9.470561 | 3.922904 | 0.000308 | 0.013934 | 0.201751 | FGR |
| 224707_at | 1.453789 | 8.133706 | 5.367765 | 2.96E-06 | 0.000737 | 4.515539 | CYSTM1 |
| 201744_s_at | 1.451991 | 3.324941 | 3.173216 | 0.002773 | 0.052496 | -1.80367 | LUM |
| 219134_at | 1.442805 | 4.700915 | 3.112655 | 0.003281 | 0.05852 | -1.95511 | ELTD1 |
| 208450_at | 1.437687 | 6.637731 | 3.824568 | 0.000416 | 0.016891 | -0.07347 | LGALS2 |
| 219622_at | 1.437457 | 5.666273 | 5.140591 | 6.28E-06 | 0.001237 | 3.813892 | RAB20 |
| 1560169_at | 1.430178 | 6.366066 | 3.705894 | 0.000594 | 0.021441 | -0.4012 | LOC101927069 |
| 204682_at | 1.422827 | 6.783369 | 3.438455 | 0.001305 | 0.033429 | -1.12031 | LTBP2 |
| 206420_at | 1.421494 | 7.733038 | 3.908774 | 0.000322 | 0.014369 | 0.162009 | IGSF6 |
| 213524_s_at | 1.420431 | 10.68044 | 3.944185 | 0.000289 | 0.013655 | 0.261729 | G0S2 |
| 236465_at | 1.413096 | 8.156624 | 3.582415 | 0.000857 | 0.026345 | -0.73671 | RNF175 |
| 203066_at | 1.409021 | 8.27328 | 3.364393 | 0.001615 | 0.037975 | -1.31428 | CHST15 |
| 212328_at | 1.408958 | 6.360789 | 4.202488 | 0.00013 | 0.008285 | 1.00069 | LIMCH1 |
| 226726_at | 1.407635 | 7.68942 | 3.561623 | 0.000911 | 0.027558 | -0.79263 | MBOAT2 |
| 213418_at | 1.405892 | 7.344852 | 3.643961 | 0.000714 | 0.023681 | -0.5702 | HSPA6 |
| 203184_at | 1.403957 | 6.057125 | 2.737382 | 0.00895 | 0.10687 | -2.85126 | FBN2 |
| 241824_at | 1.394291 | 9.454195 | 4.848617 | 1.64E-05 | 0.002231 | 2.921324 | RP11-373D23.2 |
| 1568768_s_at | 1.390583 | 5.420642 | 4.939441 | 1.22E-05 | 0.001907 | 3.197678 | BRE-AS1 |
| 1556842_at | 1.387629 | 4.12292 | 2.750322 | 0.008656 | 0.105243 | -2.82164 | LOC286087 |
| 235944_at | 1.380913 | 3.536841 | 3.188618 | 0.002657 | 0.05132 | -1.76487 | HMCN1 |
| 201739_at | 1.368135 | 10.81741 | 4.883206 | 1.46E-05 | 0.002084 | 3.026419 | SGK1 |
| 206995_x_at | 1.366878 | 6.244379 | 3.510709 | 0.001058 | 0.029671 | -0.92883 | SCARF1 |
| 207808_s_at | 1.365358 | 6.339849 | 2.879102 | 0.006175 | 0.084968 | -2.52175 | PROS1 |
| 241627_x_at | 1.363748 | 9.509994 | 3.514397 | 0.001046 | 0.029601 | -0.919 | ARHGEF40 |
| 218319_at | 1.361298 | 9.883744 | 6.247424 | 1.56E-07 | 0.00012 | 7.263132 | PELI1 |
| 201185_at | 1.359451 | 5.763263 | 3.804621 | 0.000442 | 0.017609 | -0.1289 | HTRA1 |
| 205789_at | 1.349601 | 6.616494 | 2.880528 | 0.006152 | 0.084961 | -2.51838 | CD1D |
| 234985_at | 1.346915 | 6.587606 | 3.655503 | 0.00069 | 0.02325 | -0.53882 | LDLRAD3 |
| 1559507_at | 1.346682 | 7.094312 | 3.331329 | 0.001776 | 0.040169 | -1.40011 | LOC100130357 |
| 227126_at | 1.346681 | 4.835077 | 3.692853 | 0.000618 | 0.021749 | -0.4369 | PTPRG |
| 221204_s_at | 1.345436 | 5.930176 | 3.679362 | 0.000643 | 0.022194 | -0.47378 | CRTAC1 |
| 201005_at | 1.343541 | 6.339757 | 2.999125 | 0.004476 | 0.069764 | -2.23409 | CD9 |
| 219132_at | 1.340966 | 8.963998 | 5.700051 | 9.77E-07 | 0.000365 | 5.549663 | PELI2 |
| 213716_s_at | 1.340918 | 6.440721 | 4.13054 | 0.000162 | 0.009377 | 0.792913 | SECTM1 |
| 225987_at | 1.33884 | 6.572092 | 2.361793 | 0.022754 | 0.180819 | -3.66668 | STEAP4 |
| 226111_s_at | 1.335686 | 5.99148 | 3.996724 | 0.000246 | 0.012348 | 0.410421 | ZNF385A |
| 210992_x_at | 1.334075 | 8.413909 | 4.089002 | 0.000185 | 0.010155 | 0.673622 | FCGR2C |
| 205639_at | 1.332043 | 8.274795 | 4.093687 | 0.000182 | 0.010111 | 0.687051 | AOAH |
| 207465_at | 1.330257 | 5.552662 | 3.109516 | 0.003309 | 0.058835 | -1.96291 | LOC100127886 |
| 1554999_at | 1.326825 | 6.067628 | 2.858655 | 0.006519 | 0.087899 | -2.56998 | RASGEF1B |
| 202597_at | 1.324847 | 5.346955 | 3.881772 | 0.000349 | 0.0152 | 0.086241 | IRF6 |
| 226817_at | 1.323897 | 5.115536 | 3.017633 | 0.004256 | 0.068458 | -2.18906 | DSC2 |
| 201502_s_at | 1.317358 | 11.57659 | 5.86325 | 5.65E-07 | 0.000245 | 6.059794 | NFKBIA |
| 220000_at | 1.317198 | 6.141147 | 3.292099 | 0.001985 | 0.042946 | -1.50131 | SIGLEC5 |
| 202729_s_at | 1.31677 | 6.175649 | 2.307245 | 0.025887 | 0.194445 | -3.77762 | LTBP1 |
| 226751_at | 1.316711 | 5.222492 | 4.897148 | 1.40E-05 | 0.002045 | 3.068833 | CNRIP1 |
| 201887_at | 1.316403 | 7.162872 | 3.943481 | 0.000289 | 0.013655 | 0.259745 | IL13RA1 |
| 206481_s_at | 1.316126 | 5.125395 | 3.3669 | 0.001604 | 0.037833 | -1.30775 | LDB2 |
| 232555_at | 1.308378 | 6.920535 | 3.195403 | 0.002607 | 0.050829 | -1.74774 | CREB5 |
| 1554173_at | 1.307164 | 5.860328 | 3.584926 | 0.000851 | 0.026253 | -0.72995 | CD300LB |
| 205908_s_at | 1.306986 | 4.325553 | 3.492893 | 0.001114 | 0.030479 | -0.97625 | OMD |
| 208869_s_at | 1.304922 | 8.840423 | 7.167504 | 7.10E-09 | 1.81E-05 | 10.13295 | GABARAPL1 |
| 230127_at | 1.303732 | 7.459895 | 3.83701 | 0.0004 | 0.016626 | -0.03883 | RP6-99M1.2 |
| 202388_at | 1.302878 | 11.53301 | 4.974646 | 1.08E-05 | 0.001793 | 3.30513 | RGS2 |
| 220532_s_at | 1.302299 | 5.741761 | 2.721069 | 0.009335 | 0.109506 | -2.88845 | TMEM176B |
| 1554283_at | 1.300879 | 8.073681 | 3.449462 | 0.001264 | 0.032785 | -1.09128 | CCRN4L |
| 232080_at | 1.296508 | 5.335188 | 4.170884 | 0.000143 | 0.008561 | 0.909247 | HECW2 |
| 238893_at | 1.292725 | 8.388879 | 4.122541 | 0.000166 | 0.009512 | 0.769904 | LINC00936 |
| 202988_s_at | 1.292127 | 10.23159 | 4.585135 | 3.85E-05 | 0.003839 | 2.127544 | RGS1 |
| 227697_at | 1.291324 | 10.79972 | 5.297439 | 3.74E-06 | 0.000871 | 4.297772 | SOCS3 |
| 207094_at | 1.287128 | 7.178629 | 3.969744 | 0.000267 | 0.013023 | 0.333958 | CXCR1 |
| 221724_s_at | 1.283962 | 7.630983 | 3.330672 | 0.001779 | 0.040169 | -1.40181 | CLEC4A |
| 203603_s_at | 1.278039 | 8.826483 | 4.245831 | 0.000113 | 0.007637 | 1.126535 | ZEB2 |
| 206881_s_at | 1.273402 | 6.864366 | 3.833431 | 0.000405 | 0.016648 | -0.0488 | LILRA3 |
| 226701_at | 1.273142 | 4.369251 | 3.371896 | 0.001581 | 0.037484 | -1.29474 | GJA5 |
| 235536_at | 1.272432 | 7.036539 | 6.035065 | 3.18E-07 | 0.000181 | 6.597741 | SNORD89 |
| 212099_at | 1.269918 | 9.263243 | 3.990326 | 0.000251 | 0.012477 | 0.392268 | RHOB |
| 218856_at | 1.267745 | 5.03869 | 3.609919 | 0.00079 | 0.025059 | -0.66248 | TNFRSF21 |
| 209122_at | 1.266149 | 8.940103 | 5.493356 | 1.95E-06 | 0.000578 | 4.905495 | PLIN2 |
| 228624_at | 1.26474 | 4.378928 | 2.647555 | 0.011265 | 0.121766 | -3.05414 | TMEM144 |
| 206295_at | 1.261193 | 4.842211 | 3.237698 | 0.002315 | 0.047253 | -1.64049 | IL18 |
| 226026_at | 1.259163 | 7.651808 | 5.097786 | 7.23E-06 | 0.001351 | 3.682318 | DIRC2 |
| 203725_at | 1.258204 | 9.165582 | 6.586974 | 4.97E-08 | 7.17E-05 | 8.326136 | GADD45A |
| 222717_at | 1.25205 | 7.942577 | 2.487493 | 0.016794 | 0.151024 | -3.40359 | SDPR |
| 212169_at | 1.248845 | 5.99002 | 2.947643 | 0.005143 | 0.075395 | -2.35842 | FKBP9 |
| 210095_s_at | 1.248534 | 7.439524 | 4.520424 | 4.73E-05 | 0.004419 | 1.93463 | IGFBP3 |
| 201195_s_at | 1.243127 | 9.131056 | 3.610944 | 0.000788 | 0.025059 | -0.65971 | SLC7A5 |
| 206049_at | 1.243005 | 6.885376 | 3.584067 | 0.000853 | 0.026273 | -0.73226 | SELP |
| 205922_at | 1.242616 | 9.363878 | 3.511706 | 0.001055 | 0.029671 | -0.92618 | VNN2 |
| 1556072_at | 1.240628 | 7.790866 | 4.074343 | 0.000193 | 0.010442 | 0.63164 | LINC00528 |
| 204194_at | 1.240561 | 8.716023 | 4.428619 | 6.35E-05 | 0.005267 | 1.66249 | BACH1 |
| 244620_at | 1.239938 | 6.088665 | 3.311244 | 0.00188 | 0.041544 | -1.45201 | SLC8A1-AS1 |
| 230645_at | 1.239343 | 7.109169 | 3.131815 | 0.003111 | 0.056382 | -1.90739 | FRMD3 |
| 201069_at | 1.238521 | 6.076447 | 3.611954 | 0.000786 | 0.025059 | -0.65698 | MMP2 |
| 219304_s_at | 1.237887 | 5.576573 | 2.89514 | 0.005918 | 0.08321 | -2.48376 | PDGFD |
| 240862_at | 1.235951 | 8.365473 | 4.036173 | 0.000218 | 0.011398 | 0.522627 | RASGRP4 |
| 213792_s_at | 1.235613 | 8.991858 | 3.439735 | 0.0013 | 0.033429 | -1.11693 | INSR |
| 213316_at | 1.235277 | 4.539905 | 3.290697 | 0.001993 | 0.043049 | -1.50491 | KIAA1462 |
| 201506_at | 1.234415 | 7.926184 | 2.662852 | 0.010835 | 0.119179 | -3.01993 | TGFBI |
| 223767_at | 1.233083 | 4.291049 | 3.714072 | 0.00058 | 0.021135 | -0.37878 | GPR84 |
| 228790_at | 1.230529 | 5.014577 | 3.987786 | 0.000253 | 0.012518 | 0.385066 | FAM110B |
| 205040_at | 1.222949 | 6.401127 | 3.086632 | 0.003525 | 0.061399 | -2.01963 | ORM1 |
| 222760_at | 1.218144 | 5.951305 | 3.995599 | 0.000247 | 0.012362 | 0.407228 | ZNF703 |
| 214983_at | 1.216699 | 5.895668 | 2.089906 | 0.042536 | 0.252223 | -4.19937 | TTTY15 |
| 212651_at | 1.215438 | 4.694421 | 2.636616 | 0.011582 | 0.123304 | -3.07852 | RHOBTB1 |
| 203680_at | 1.214924 | 6.159541 | 2.255355 | 0.02922 | 0.207601 | -3.8813 | PRKAR2B |
| 209047_at | 1.211901 | 5.607598 | 2.700723 | 0.009836 | 0.112938 | -2.93463 | AQP1 |
| 233236_at | 1.209932 | 7.454566 | 3.152497 | 0.002938 | 0.054369 | -1.85568 | TSPAN16 |
| 202620_s_at | 1.208342 | 4.733594 | 2.646452 | 0.011297 | 0.121947 | -3.05661 | PLOD2 |
| 206390_x_at | 1.205946 | 9.670286 | 2.60006 | 0.0127 | 0.129057 | -3.15948 | PF4 |
| 225056_at | 1.205844 | 8.015689 | 3.663527 | 0.000674 | 0.022916 | -0.51697 | SIPA1L2 |
| 223344_s_at | 1.203432 | 6.443671 | 2.556335 | 0.014167 | 0.137331 | -3.25523 | MS4A7 |
| 226436_at | 1.203231 | 6.054226 | 2.217168 | 0.031912 | 0.217177 | -3.95641 | RASSF4 |
| 217473_x_at | 1.198183 | 8.831102 | 4.176392 | 0.000141 | 0.008561 | 0.925163 | SLC11A1 |
| 1560007_at | 1.19697 | 5.613212 | 2.740301 | 0.008883 | 0.106529 | -2.84459 | LOC645984 |
| 201160_s_at | 1.193808 | 8.886968 | 5.443558 | 2.30E-06 | 0.000639 | 4.750723 | YBX3 |
| 206464_at | 1.19283 | 4.751155 | 5.222328 | 4.79E-06 | 0.001026 | 4.065721 | BMX |
| 228080_at | 1.190103 | 5.539273 | 3.52659 | 0.00101 | 0.029378 | -0.88646 | LAYN |
| 225582_at | 1.189353 | 8.231262 | 6.041674 | 3.11E-07 | 0.000181 | 6.618445 | ITPRIP |
| 204115_at | 1.18589 | 8.745423 | 2.684805 | 0.010245 | 0.115423 | -2.97059 | GNG11 |
| 224277_at | 1.185839 | 4.691798 | 2.801707 | 0.007572 | 0.096472 | -2.7031 | MOP-1 |
| 206177_s_at | 1.185281 | 5.963746 | 2.671383 | 0.010602 | 0.117842 | -3.00079 | ARG1 |
| 205230_at | 1.185236 | 4.498187 | 2.995979 | 0.004514 | 0.07002 | -2.24173 | RPH3A |
| 205349_at | 1.185177 | 6.719298 | 4.791326 | 1.97E-05 | 0.00256 | 2.747678 | GNA15 |
| 235458_at | 1.181533 | 7.913254 | 3.084647 | 0.003544 | 0.06159 | -2.02454 | HAVCR2 |
| 214511_x_at | 1.181127 | 6.255532 | 2.492391 | 0.016593 | 0.150457 | -3.39314 | FCGR1B |
| 45297_at | 1.181032 | 5.795299 | 3.931527 | 0.0003 | 0.013831 | 0.226037 | EHD2 |
| 217865_at | 1.180386 | 9.197189 | 3.76758 | 0.000494 | 0.018991 | -0.23147 | RNF130 |
| 222496_s_at | 1.17991 | 9.17934 | 3.020228 | 0.004226 | 0.068246 | -2.18273 | RBM47 |
| 238423_at | 1.176843 | 9.620972 | 6.10054 | 2.55E-07 | 0.000168 | 6.802872 | SYTL3 |
| 222453_at | 1.176813 | 5.445717 | 3.405118 | 0.001437 | 0.035361 | -1.20791 | CYBRD1 |
| 204285_s_at | 1.173592 | 9.091467 | 3.932988 | 0.000299 | 0.013799 | 0.230155 | PMAIP1 |
| 218793_s_at | 1.170607 | 6.928028 | 3.550463 | 0.000942 | 0.028279 | -0.82257 | SCML1 |
| 243201_at | 1.166993 | 7.829707 | 3.942215 | 0.000291 | 0.013677 | 0.256173 | RPL36A |
| 204627_s_at | 1.162281 | 6.018209 | 2.236911 | 0.030494 | 0.211659 | -3.9177 | ITGB3 |
| 201200_at | 1.162028 | 9.035852 | 4.500446 | 5.05E-05 | 0.004498 | 1.875253 | CREG1 |
| 207113_s_at | 1.162024 | 8.418659 | 2.668217 | 0.010688 | 0.118453 | -3.0079 | TNF |
| 231925_at | 1.161809 | 4.885005 | 2.646025 | 0.011309 | 0.121994 | -3.05756 | RP11-38P22.2 |
| 208018_s_at | 1.158224 | 7.579343 | 2.833141 | 0.006973 | 0.09149 | -2.62984 | HCK |
| 203104_at | 1.157317 | 5.579155 | 2.44209 | 0.01876 | 0.161356 | -3.4998 | CSF1R |
| 230972_at | 1.154477 | 6.770365 | 3.365797 | 0.001609 | 0.037871 | -1.31063 | ANKRD9 |
| 204589_at | 1.153755 | 6.073497 | 3.934697 | 0.000297 | 0.013778 | 0.234971 | NUAK1 |
| 226188_at | 1.153652 | 6.539116 | 2.394908 | 0.021022 | 0.172307 | -3.59837 | LGALSL |
| 218865_at | 1.153335 | 5.879056 | 2.875218 | 0.006239 | 0.085509 | -2.53093 | 1-Mar |
| 203708_at | 1.152264 | 10.86533 | 8.316358 | 1.61E-10 | 1.75E-06 | 13.62745 | PDE4B |
| 218345_at | 1.151796 | 5.881056 | 2.492539 | 0.016587 | 0.150457 | -3.39282 | TMEM176A |
| 203868_s_at | 1.149387 | 4.423409 | 3.477108 | 0.001167 | 0.031249 | -1.01814 | VCAM1 |
| 230505_at | 1.14915 | 8.598837 | 5.629807 | 1.24E-06 | 0.000412 | 5.330464 | LOC145474 |
| 202974_at | 1.149149 | 7.922368 | 3.937008 | 0.000295 | 0.013754 | 0.241487 | MPP1 |
| 210321_at | 1.14739 | 8.684491 | 2.577041 | 0.013454 | 0.133522 | -3.21003 | GZMH |
| 203853_s_at | 1.146331 | 7.704241 | 4.780961 | 2.04E-05 | 0.002601 | 2.71632 | GAB2 |
| 202241_at | 1.14402 | 10.38959 | 4.401281 | 6.92E-05 | 0.005574 | 1.581821 | TRIB1 |
| 224657_at | 1.141785 | 6.480783 | 3.834744 | 0.000403 | 0.016626 | -0.04514 | ERRFI1 |
| 205312_at | 1.140696 | 7.47937 | 3.738188 | 0.000539 | 0.020086 | -0.31251 | SPI1 |
| 231411_at | 1.138307 | 5.365791 | 3.966898 | 0.000269 | 0.013049 | 0.325904 | LHFP |
| 205936_s_at | 1.136203 | 5.944846 | 3.446876 | 0.001274 | 0.032992 | -1.0981 | HK3 |
| 1556185_a_at | 1.135893 | 3.876444 | 2.386906 | 0.021429 | 0.17419 | -3.61495 | CTB-167B5.2 |
| 227044_at | 1.1346 | 8.416696 | 3.067868 | 0.003711 | 0.063324 | -2.06594 | CTA-29F11.1 |
| 212531_at | 1.134153 | 6.556763 | 2.273383 | 0.028021 | 0.202769 | -3.84549 | LCN2 |
| 221345_at | 1.133776 | 6.128156 | 2.999115 | 0.004476 | 0.069764 | -2.23411 | FFAR2 |
| 1554057_at | 1.131319 | 7.339372 | 2.431008 | 0.019271 | 0.163971 | -3.52308 | ASH1L-AS1 |
| 218963_s_at | 1.130964 | 6.628562 | 2.806469 | 0.007478 | 0.095937 | -2.69204 | KRT23 |
| 210184_at | 1.130212 | 9.645002 | 3.306548 | 0.001905 | 0.041889 | -1.46412 | ITGAX |
| 219593_at | 1.129773 | 6.276084 | 3.521466 | 0.001025 | 0.0294 | -0.90014 | SLC15A3 |
| 206584_at | 1.129603 | 10.07226 | 4.172026 | 0.000143 | 0.008561 | 0.912545 | LY96 |
| 207111_at | 1.128224 | 6.319678 | 3.822148 | 0.000419 | 0.01695 | -0.08021 | EMR1 |
| 206676_at | 1.128203 | 4.619882 | 2.176464 | 0.035022 | 0.227951 | -4.03536 | CEACAM8 |
| 202018_s_at | 1.127426 | 6.000841 | 2.2535 | 0.029346 | 0.208034 | -3.88497 | LTF |
| 222162_s_at | 1.125575 | 4.657609 | 3.734798 | 0.000545 | 0.020088 | -0.32184 | ADAMTS1 |
| 219890_at | 1.123523 | 4.363022 | 2.494055 | 0.016526 | 0.150172 | -3.38958 | CLEC5A |
| 217897_at | 1.12245 | 5.62937 | 3.63559 | 0.000732 | 0.023886 | -0.59294 | FXYD6 |
| 201118_at | 1.121408 | 7.925077 | 4.368389 | 7.69E-05 | 0.005978 | 1.484992 | PGD |
| 201261_x_at | 1.119986 | 6.179341 | 4.388076 | 7.22E-05 | 0.005759 | 1.542915 | BGN |
| 232629_at | 1.119763 | 7.766677 | 3.103034 | 0.003369 | 0.059538 | -1.979 | PROK2 |
| 209524_at | 1.118147 | 6.620273 | 3.226301 | 0.00239 | 0.048288 | -1.66947 | HDGFRP3 |
| 227180_at | 1.117177 | 4.719256 | 2.124202 | 0.039404 | 0.242553 | -4.13503 | ELOVL7 |
| 212820_at | 1.115057 | 8.302574 | 2.55876 | 0.014082 | 0.136749 | -3.24995 | DMXL2 |
| 1554443_s_at | 1.113733 | 8.093871 | 2.979147 | 0.004724 | 0.07169 | -2.2825 | BEST1 |
| 204249_s_at | 1.112281 | 6.844802 | 2.967987 | 0.004869 | 0.073062 | -2.30945 | LMO2 |
| 203922_s_at | 1.101686 | 7.525949 | 2.91216 | 0.005655 | 0.080355 | -2.44329 | CYBB |
| 203002_at | 1.100691 | 5.253974 | 3.493553 | 0.001112 | 0.030479 | -0.97449 | AMOTL2 |
| 216951_at | 1.099725 | 3.851889 | 2.870598 | 0.006316 | 0.086072 | -2.54184 | FCGR1A |
| 218739_at | 1.097833 | 7.44945 | 4.132058 | 0.000162 | 0.009358 | 0.797281 | ABHD5 |
| 200919_at | 1.097518 | 8.4256 | 5.688063 | 1.02E-06 | 0.000373 | 5.512236 | PHC2 |
| 203665_at | 1.095259 | 5.338965 | 3.085428 | 0.003536 | 0.061507 | -2.02261 | HMOX1 |
| 218831_s_at | 1.094688 | 8.305556 | 4.185173 | 0.000137 | 0.008399 | 0.950556 | FCGRT |
| 206942_s_at | 1.094539 | 7.615729 | 2.972264 | 0.004813 | 0.072659 | -2.29913 | PMCH |
| 235607_at | 1.09181 | 7.597597 | 2.674399 | 0.010521 | 0.117321 | -2.99401 | LOC101928963 |
| 204894_s_at | 1.085284 | 3.829794 | 3.63624 | 0.000731 | 0.02388 | -0.59117 | AOC3 |
| 205645_at | 1.0845 | 6.815873 | 3.927672 | 0.000304 | 0.013889 | 0.215176 | REPS2 |
| 210724_at | 1.080708 | 5.970252 | 3.128357 | 0.003141 | 0.056687 | -1.91602 | EMR3 |
| 231771_at | 1.078985 | 5.399267 | 3.299179 | 0.001946 | 0.042474 | -1.4831 | GJB6 |
| 202644_s_at | 1.077709 | 12.12723 | 6.665993 | 3.81E-08 | 6.34E-05 | 8.573061 | TNFAIP3 |
| 1559489_a_at | 1.07581 | 5.609144 | 2.924157 | 0.005477 | 0.078593 | -2.41467 | LINC01366 |
| 1552553_a_at | 1.068235 | 6.86541 | 2.823365 | 0.007154 | 0.093047 | -2.65269 | NLRC4 |
| 1560625_s_at | 1.067833 | 5.983517 | 4.20571 | 0.000128 | 0.008285 | 1.010028 | CATIP-AS1 |
| 202391_at | 1.067159 | 11.30413 | 4.267828 | 0.000106 | 0.007262 | 1.190593 | BASP1 |
| 202393_s_at | 1.066814 | 8.98523 | 3.988275 | 0.000252 | 0.012518 | 0.386452 | KLF10 |
| 228412_at | 1.060047 | 6.014643 | 2.63772 | 0.011549 | 0.123081 | -3.07607 | LOC643072 |
| 219584_at | 1.057391 | 5.0627 | 3.46218 | 0.001218 | 0.031941 | -1.05767 | PLA1A |
| 211423_s_at | 1.056791 | 8.721201 | 3.699067 | 0.000606 | 0.021554 | -0.4199 | SC5D |
| 233072_at | 1.055212 | 8.708942 | 3.541434 | 0.000967 | 0.02868 | -0.84676 | NTNG2 |
| 201328_at | 1.053998 | 8.758677 | 3.523576 | 0.001019 | 0.029393 | -0.89451 | ETS2 |
| 218901_at | 1.052105 | 4.243272 | 2.82954 | 0.007039 | 0.091991 | -2.63826 | PLSCR4 |
| 210176_at | 1.051898 | 8.1181 | 3.474582 | 0.001175 | 0.031342 | -1.02484 | TLR1 |
| 201489_at | 1.051533 | 9.160266 | 4.045207 | 0.000212 | 0.011287 | 0.548388 | PPIF |
| 227554_at | 1.051369 | 3.3406 | 3.623665 | 0.000759 | 0.024451 | -0.62527 | MAGI2-AS3 |
| 206283_s_at | 1.050547 | 6.769239 | 2.134447 | 0.038509 | 0.240044 | -4.11564 | TAL1 |
| 237340_at | 1.049584 | 4.809042 | 3.205877 | 0.002531 | 0.04988 | -1.72126 | SLC26A8 |
| 213138_at | 1.048955 | 7.688906 | 5.129893 | 6.51E-06 | 0.001258 | 3.780988 | ARID5A |
| 240747_at | 1.046064 | 4.643138 | 3.267535 | 0.002128 | 0.044903 | -1.56432 | LOC102724587 |
| 202446_s_at | 1.042187 | 8.311472 | 3.391641 | 0.001494 | 0.036177 | -1.24319 | PLSCR1 |
| 206440_at | 1.041364 | 5.871533 | 3.479391 | 0.001159 | 0.031249 | -1.01209 | LIN7A |
| 202449_s_at | 1.040195 | 7.812892 | 4.332197 | 8.62E-05 | 0.006307 | 1.378747 | RXRA |
| 219714_s_at | 1.038997 | 4.351647 | 2.454695 | 0.018194 | 0.158805 | -3.47323 | CACNA2D3 |
| 210793_s_at | 1.038454 | 9.369369 | 5.44155 | 2.32E-06 | 0.000639 | 4.744486 | NUP98 |
| 228037_at | 1.038437 | 5.03998 | 3.318094 | 0.001844 | 0.0411 | -1.43433 | RARA-AS1 |
| 35776_at | 1.035676 | 7.018407 | 3.095706 | 0.003438 | 0.060473 | -1.99717 | ITSN1 |
| 228918_at | 1.032906 | 6.947803 | 3.850544 | 0.000384 | 0.016251 | -0.00109 | SLC43A2 |
| 216336_x_at | 1.030684 | 9.037464 | 4.708282 | 2.58E-05 | 0.003025 | 2.496972 | MT1E |
| 211732_x_at | 1.030562 | 6.412875 | 3.306735 | 0.001904 | 0.041889 | -1.46363 | HNMT |
| 204204_at | 1.025509 | 8.602852 | 3.654753 | 0.000692 | 0.023266 | -0.54086 | SLC31A2 |
| 201525_at | 1.024803 | 4.340536 | 4.113603 | 0.000171 | 0.009656 | 0.744212 | APOD |
| 210401_at | 1.024339 | 6.120681 | 2.847851 | 0.006708 | 0.089331 | -2.59538 | P2RX1 |
| 207387_s_at | 1.023238 | 7.615441 | 3.151257 | 0.002948 | 0.054424 | -1.85878 | GK |
| 213281_at | 1.020151 | 10.65263 | 3.4665 | 0.001203 | 0.031697 | -1.04624 | JUN |
| 212070_at | 1.018425 | 7.326896 | 2.419905 | 0.019795 | 0.16614 | -3.54633 | GPR56 |
| 219298_at | 1.018415 | 6.031384 | 4.823607 | 1.78E-05 | 0.002347 | 2.845453 | ECHDC3 |
| 208067_x_at | 1.01766 | 7.829748 | 3.512536 | 0.001052 | 0.029671 | -0.92396 | UTY |
| 213624_at | 1.017178 | 5.390865 | 3.361759 | 0.001628 | 0.038099 | -1.32114 | SMPDL3A |
| 209821_at | 1.015986 | 3.417633 | 2.563816 | 0.013906 | 0.135829 | -3.23893 | IL33 |
| 201615_x_at | 1.015644 | 7.350273 | 3.8401 | 0.000397 | 0.01655 | -0.03022 | CALD1 |
| 220416_at | 1.012542 | 4.549841 | 2.237162 | 0.030476 | 0.211659 | -3.91721 | ATP8B4 |
| 201798_s_at | 1.008536 | 5.194648 | 2.744412 | 0.008789 | 0.10615 | -2.83518 | MYOF |
| 210225_x_at | 1.007891 | 8.053783 | 3.523953 | 0.001018 | 0.029393 | -0.8935 | LILRB3 |
| 206697_s_at | 1.007132 | 6.700364 | 3.379315 | 0.001548 | 0.037034 | -1.27539 | HP |
| 1569767_at | 1.007014 | 6.451309 | 2.93424 | 0.005331 | 0.077216 | -2.39055 | LOC101927131 |
| 204970_s_at | 1.00543 | 6.986743 | 4.034529 | 0.000219 | 0.011398 | 0.517941 | MAFG |
| 225154_at | 1.005405 | 10.044 | 4.455363 | 5.83E-05 | 0.004989 | 1.741577 | SYAP1 |
| 206414_s_at | 1.005001 | 6.355593 | 2.583885 | 0.013226 | 0.132046 | -3.19504 | ASAP2 |
| 231600_at | 1.003039 | 3.918286 | 2.236544 | 0.03052 | 0.211659 | -3.91842 | CLEC12B |
| 210004_at | 1.001893 | 3.997779 | 2.386824 | 0.021434 | 0.17419 | -3.61512 | OLR1 |
| 228240_at | -1.00239 | 8.645592 | -5.24386 | 4.47E-06 | 0.000987 | 4.132188 | AGAP1 |
| 235874_at | -1.00401 | 5.559568 | -2.63442 | 0.011646 | 0.123755 | -3.0834 | PRSS35 |
| 235320_at | -1.00724 | 6.420411 | -3.58876 | 0.000841 | 0.026173 | -0.71962 | ARL6 |
| 214061_at | -1.01042 | 8.481515 | -4.67236 | 2.90E-05 | 0.003242 | 2.388903 | TBC1D31 |
| 236394_at | -1.0139 | 7.576469 | -4.16585 | 0.000145 | 0.008653 | 0.894697 | A2MP1 |
| 1557733_a_at | -1.01564 | 9.842751 | -5.41173 | 2.56E-06 | 0.000667 | 4.651907 | CHRM3-AS2 |
| 236194_at | -1.01742 | 7.800822 | -3.67483 | 0.000652 | 0.022388 | -0.48614 | RP11-53O19.3 |
| 223377_x_at | -1.01915 | 9.067266 | -2.92056 | 0.00553 | 0.07909 | -2.42325 | CISH |
| 229088_at | -1.02575 | 5.641505 | -2.52213 | 0.015422 | 0.144569 | -3.32932 | ENPP1 |
| 206073_at | -1.03295 | 8.544719 | -2.2787 | 0.027676 | 0.201726 | -3.83489 | COLQ |
| 1552563_a_at | -1.03336 | 4.785722 | -3.11925 | 0.003221 | 0.057701 | -1.93871 | AF131215.4 |
| 206978_at | -1.03414 | 10.24356 | -3.9053 | 0.000325 | 0.014462 | 0.15225 | CCR2 |
| 226977_at | -1.03835 | 8.755678 | -3.68779 | 0.000627 | 0.021902 | -0.45074 | IGIP |
| 213848_at | -1.04079 | 9.359894 | -5.44518 | 2.29E-06 | 0.000639 | 4.755746 | DUSP7 |
| 203476_at | -1.04429 | 7.029012 | -3.94397 | 0.000289 | 0.013655 | 0.261116 | TPBG |
| 235736_at | -1.04625 | 4.972651 | -3.89313 | 0.000338 | 0.014887 | 0.118069 | SMKR1 |
| 242557_at | -1.05513 | 8.074834 | -3.07903 | 0.003599 | 0.062051 | -2.03842 | ZNRD1-AS1 |
| 232165_at | -1.05836 | 8.291254 | -2.42886 | 0.019371 | 0.164374 | -3.52759 | EPPK1 |
| 212771_at | -1.05894 | 8.600143 | -4.14803 | 0.000154 | 0.008999 | 0.843283 | FAM171A1 |
| 210694_s_at | -1.0634 | 8.23144 | -3.77518 | 0.000483 | 0.018727 | -0.21045 | MID1 |
| 229810_at | -1.06461 | 6.659717 | -2.57502 | 0.013522 | 0.133702 | -3.21445 | RSPH3 |
| 1555040_at | -1.06494 | 4.651068 | -3.62891 | 0.000747 | 0.024218 | -0.61107 | LINC00612 |
| 204129_at | -1.07971 | 6.224684 | -5.49961 | 1.91E-06 | 0.000574 | 4.924951 | BCL9 |
| 230720_at | -1.08659 | 5.296007 | -2.99625 | 0.004511 | 0.07002 | -2.24107 | RNF182 |
| 1560550_at | -1.10116 | 7.892581 | -2.3292 | 0.024582 | 0.188048 | -3.7332 | LOC101927722 |
| 213861_s_at | -1.10357 | 7.458744 | -4.48584 | 5.29E-05 | 0.004599 | 1.831887 | METTL21B |
| 1560147_at | -1.10401 | 6.359773 | -4.64582 | 3.16E-05 | 0.003425 | 2.30923 | WDR86-AS1 |
| 242761_s_at | -1.10547 | 9.569776 | -3.74954 | 0.000521 | 0.01963 | -0.28124 | ZNF420 |
| 213994_s_at | -1.11248 | 7.894844 | -3.3912 | 0.001496 | 0.036177 | -1.24435 | SPON1 |
| 1564139_at | -1.1193 | 9.763167 | -4.2967 | 9.64E-05 | 0.006825 | 1.274856 | A2M-AS1 |
| 230927_at | -1.11957 | 5.315312 | -4.19665 | 0.000132 | 0.008285 | 0.983787 | CTD-3025N20.3 |
| 209686_at | -1.12465 | 5.635668 | -2.78926 | 0.007822 | 0.098712 | -2.73194 | S100B |
| 219532_at | -1.12953 | 8.106876 | -3.12203 | 0.003197 | 0.057354 | -1.93178 | ELOVL4 |
| 1552912_a_at | -1.14588 | 6.436192 | -2.63004 | 0.011776 | 0.124333 | -3.09315 | IL23R |
| 209975_at | -1.17277 | 7.722248 | -3.15009 | 0.002958 | 0.054438 | -1.8617 | CYP2E1 |
| 228329_at | -1.17589 | 6.698376 | -2.87941 | 0.00617 | 0.084968 | -2.52102 | DAB1 |
| 228806_at | -1.17761 | 8.462773 | -4.16155 | 0.000147 | 0.008744 | 0.882294 | RORC |
| 209815_at | -1.22242 | 9.179473 | -3.84859 | 0.000387 | 0.016284 | -0.00654 | PTCH1 |
| 227626_at | -1.22699 | 8.284423 | -3.9198 | 0.000311 | 0.014008 | 0.193028 | PAQR8 |
| 239680_at | -1.23439 | 6.485005 | -4.28521 | 0.0001 | 0.006943 | 1.241291 | WDR76 |
| 1555370_a_at | -1.249 | 9.598381 | -4.15237 | 0.000152 | 0.008914 | 0.855796 | CAMTA1 |
| 238881_at | -1.27701 | 7.471711 | -4.17211 | 0.000143 | 0.008561 | 0.912799 | RP4-714D9.5 |
| 228434_at | -1.29337 | 7.106618 | -3.82931 | 0.00041 | 0.016743 | -0.06028 | BTNL9 |
| 231798_at | -1.31726 | 6.808507 | -3.43479 | 0.001319 | 0.033643 | -1.12995 | NOG |
| 220144_s_at | -1.3284 | 6.898729 | -4.90718 | 1.35E-05 | 0.001993 | 3.099377 | ANKEF1 |
| 1562245_a_at | -1.36221 | 5.892419 | -4.19946 | 0.000131 | 0.008285 | 0.991912 | ZNF578 |
| 218975_at | -1.71256 | 8.37015 | -4.11607 | 0.00017 | 0.009654 | 0.751309 | COL5A3 |
| 231592_at | -2.86401 | 6.966298 | -2.90756 | 0.005725 | 0.081108 | -2.45423 | TSIX |
| 224588_at | -3.33123 | 7.140488 | -2.91799 | 0.005568 | 0.079426 | -2.42939 | XIST |

Supplementary table 2: 471 significantly up-and 49 down-regulated genes
