# Supplementary material for: Identification and validation of senescence-related genes in circulating endothelial cells of patients with acute myocardial infarction
Source: Front Cardiovasc Med. 2022 Dec 13;9:1057985. doi: 10.3389/fcvm.2022.1057985 (PMC9792765; doi:10.3389/fcvm.2022.1057985)
Supplement: Supplementary Table 3 — One hundred and forty-eight miRNAs of 3 diagnostic genes. [file Table_3.DOCX]

| ID | Accession | Target | TargetID | Experiment | Literature |
| --- | --- | --- | --- | --- | --- |
| hsa-let-7e-5p | MIMAT0000066 | MMP9 | 4318 | Luciferase reporter assay//qRT-PCR//Western blot | 24503540 |
| hsa-mir-18a-5p | MIMAT0000072 | ETS2 | 2114 | CLASH | 23622248 |
| hsa-mir-21-5p | MIMAT0000076 | BCL6 | 604 | Luciferase reporter assay | 23416424 |
| hsa-mir-21-5p | MIMAT0000076 | MMP9 | 4318 | qRT-PCR | 19435867 |
| hsa-mir-29b-3p | MIMAT0000100 | MMP9 | 4318 | Luciferase reporter assay//qRT-PCR | 23354167 |
| hsa-mir-199a-5p | MIMAT0000231 | ETS2 | 2114 | Microarray//Northern blot | 16331254 |
| hsa-mir-199a-3p | MIMAT0000232 | ETS2 | 2114 | Microarray//Northern blot | 16331254 |
| hsa-mir-10a-5p | MIMAT0000253 | BCL6 | 604 | Luciferase reporter assay//Western blot | 26590574 |
| hsa-mir-187-3p | MIMAT0000262 | BCL6 | 604 | Luciferase reporter assay | 26845350\|27073562 |
| hsa-mir-204-5p | MIMAT0000265 | MMP9 | 4318 | Luciferase reporter assay//Western blot | 28280358 |
| hsa-mir-205-5p | MIMAT0000266 | BCL6 | 604 | Luciferase reporter assay//Western blot | 22870299 |
| hsa-mir-211-5p | MIMAT0000268 | MMP9 | 4318 | Immunoblot//Immunohistochemistry//qRT-PCR | 23183822 |
| hsa-mir-218-5p | MIMAT0000275 | ETS2 | 2114 | Sequencing | 20371350 |
| hsa-mir-15b-5p | MIMAT0000417 | MMP9 | 4318 | qRT-PCR//Western blot | 25901555 |
| hsa-mir-30b-5p | MIMAT0000420 | BCL6 | 604 | Luciferase reporter assay//qRT-PCR//Western blot | 22387553 |
| hsa-mir-124-3p | MIMAT0000422 | BCL6 | 604 | Microarray | 18668037 |
| hsa-mir-132-3p | MIMAT0000426 | MMP9 | 4318 | Luciferase reporter assay | 26319558 |
| hsa-mir-143-3p | MIMAT0000435 | MMP9 | 4318 | qRT-PCR//Western blot | 26722463 |
| hsa-mir-9-5p | MIMAT0000441 | BCL6 | 604 | Luciferase reporter assay | 19956200 |
| hsa-mir-9-5p | MIMAT0000441 | MMP9 | 4318 | qRT-PCR | 21931274 |
| hsa-mir-9-3p | MIMAT0000442 | MMP9 | 4318 | Western blot | 28413458 |
| hsa-mir-127-3p | MIMAT0000446 | BCL6 | 604 | Immunoblot//Luciferase reporter assay//Microarray//Northern blot//qRT-PCR//Western blot | 17581274\|16766263\|24282530 |
| hsa-mir-149-5p | MIMAT0000450 | ETS2 | 2114 | CLASH | 23622248 |
| hsa-mir-320a | MIMAT0000510 | MMP9 | 4318 | In situ hybridization//Microarray | 25468268 |
| hsa-mir-155-5p | MIMAT0000646 | BCL6 | 604 | Luciferase reporter assay//qRT-PCR//Western blot | 23041630\|26986233 |
| hsa-mir-302a-5p | MIMAT0000683 | MMP9 | 4318 | ELISA//Western blot | 26191138 |
| hsa-mir-338-3p | MIMAT0000763 | MMP9 | 4318 | qRT-PCR//Western blot | 21671467 |
| hsa-mir-339-5p | MIMAT0000764 | BCL6 | 604 | Immunohistochemistry//Luciferase reporter assay//qRT-PCR//Western blot | 20932331\|24917186 |
| hsa-mir-335-5p | MIMAT0000765 | BCL6 | 604 | Microarray | 18185580 |
| hsa-mir-133b | MIMAT0000770 | MMP9 | 4318 | Luciferase reporter assay | 24714873 |
| hsa-mir-346 | MIMAT0000773 | BCL6 | 604 | Flow//Luciferase reporter assay//qRT-PCR//Western blot | 25666935 |
| hsa-mir-196b-5p | MIMAT0001080 | ETS2 | 2114 | ChIP-seq//Luciferase reporter assay//qRT-PCR//Western blot | 22298639 |
| hsa-mir-451a | MIMAT0001631 | MMP9 | 4318 | qRT-PCR//Western blot | 20816946 |
| hsa-mir-410-3p | MIMAT0002171 | ETS2 | 2114 | PAR-CLIP | 20371350 |
| hsa-mir-491-5p | MIMAT0002807 | MMP9 | 4318 | Immunohistochemistry//Luciferase reporter assay//Microarray//qRT-PCR//Western blot | 21831363\|23725476\|27023472 |
| hsa-mir-524-5p | MIMAT0002849 | MMP9 | 4318 | qRT-PCR//Western blot | 26998102 |
| hsa-mir-455-5p | MIMAT0003150 | ETS2 | 2114 | PAR-CLIP | 21572407 |
| hsa-mir-544a | MIMAT0003164 | BCL6 | 604 | Luciferase reporter assay//qRT-PCR//Western blot | 27186677 |
| hsa-mir-582-5p | MIMAT0003247 | ETS2 | 2114 | PAR-CLIP | 20371350 |
| hsa-mir-587 | MIMAT0003253 | ETS2 | 2114 | PAR-CLIP | 21572407 |
| hsa-mir-607 | MIMAT0003275 | ETS2 | 2114 | PAR-CLIP | 20371350 |
| hsa-mir-338-5p | MIMAT0004701 | ETS2 | 2114 | PAR-CLIP | 20371350 |
| hsa-mir-892b | MIMAT0004918 | MMP9 | 4318 | Immunoblot | 27573859 |
| hsa-mir-877-5p | MIMAT0004949 | ETS2 | 2114 | CLASH | 23622248 |
| hsa-mir-944 | MIMAT0004987 | ETS2 | 2114 | PAR-CLIP | 20371350 |
| hsa-mir-205-3p | MIMAT0009197 | ETS2 | 2114 | PAR-CLIP | 20371350 |
| hsa-mir-3646 | MIMAT0018065 | ETS2 | 2114 | PAR-CLIP | 21572407 |
| hsa-mir-3662 | MIMAT0018083 | ETS2 | 2114 | PAR-CLIP | 21572407 |
| hsa-mir-3671 | MIMAT0018094 | ETS2 | 2114 | PAR-CLIP | 20371350 |
| hsa-mir-548aa | MIMAT0018447 | ETS2 | 2114 | PAR-CLIP | 21572407 |
| hsa-mir-548ap-3p | MIMAT0021038 | ETS2 | 2114 | PAR-CLIP | 21572407 |
| hsa-mir-548as-3p | MIMAT0022268 | ETS2 | 2114 | PAR-CLIP | 21572407 |
| hsa-mir-548at-3p | MIMAT0022278 | ETS2 | 2114 | PAR-CLIP | 21572407 |
| hsa-mir-548aw | MIMAT0022471 | ETS2 | 2114 | PAR-CLIP | 21572407 |
| hsa-mir-548g-5p | MIMAT0022722 | ETS2 | 2114 | PAR-CLIP | 21572407 |
| hsa-mir-548t-3p | MIMAT0022730 | ETS2 | 2114 | PAR-CLIP | 21572407 |
| hsa-mir-548x-5p | MIMAT0022733 | ETS2 | 2114 | PAR-CLIP | 21572407 |
| hsa-mir-548aj-5p | MIMAT0022739 | ETS2 | 2114 | PAR-CLIP | 21572407 |
| hsa-mir-548ay-3p | MIMAT0025453 | ETS2 | 2114 | PAR-CLIP | 21572407 |
| hsa-mir-133a-5p | MIMAT0026478 | MMP9 | 4318 | Luciferase reporter assay//qRT-PCR//Western blot | 25607810 |
| hsa-mir-190a-3p | MIMAT0026482 | ETS2 | 2114 | PAR-CLIP | 20371350 |
| hsa-mir-1468-3p | MIMAT0026638 | ETS2 | 2114 | PAR-CLIP | 21572407 |
| hsa-mir-942-3p | MIMAT0026734 | MMP9 | 4318 | qRT-PCR//Western blot | 24475095 |
| hsa-mir-548f-5p | MIMAT0026739 | ETS2 | 2114 | PAR-CLIP | 21572407 |
| hsa-mir-203a-5p | MIMAT0031890 | MMP9 | 4318 | qRT-PCR | 26278219 |
| hsa-mir-103a-3p | MIMAT0000101 | MMP9 | 4318 | HITS-CLIP | tarbase |
| hsa-mir-107 | MIMAT0000104 | MMP9 | 4318 | HITS-CLIP | tarbase |
| hsa-mir-124-3p | MIMAT0000422 | MMP9 | 4318 | Western Blot | tarbase |
| hsa-mir-128-3p | MIMAT0000424 | MMP9 | 4318 | Western Blot | tarbase |
| hsa-mir-138-5p | MIMAT0000430 | MMP9 | 4318 | Microarrays | tarbase |
| hsa-mir-141-3p | MIMAT0000432 | MMP9 | 4318 | Western Blot | tarbase |
| hsa-mir-145-5p | MIMAT0000437 | MMP9 | 4318 | Western Blot, qPCR, Luciferase Reporter Assay, ELISA | tarbase |
| hsa-mir-195-5p | MIMAT0000461 | MMP9 | 4318 | qPCR | tarbase |
| hsa-mir-200a-3p | MIMAT0000682 | MMP9 | 4318 | Western Blot | tarbase |
| hsa-mir-218-5p | MIMAT0000275 | MMP9 | 4318 | Western Blot | tarbase |
| hsa-mir-223-3p | MIMAT0000280 | MMP9 | 4318 | Western Blot | tarbase |
| hsa-mir-23b-3p | MIMAT0000418 | MMP9 | 4318 | qPCR | tarbase |
| hsa-mir-34a-5p | MIMAT0000255 | MMP9 | 4318 | qPCR | tarbase |
| hsa-mir-376c-3p | MIMAT0000720 | MMP9 | 4318 | Microarrays, qPCR | tarbase |
| hsa-mir-377-3p | MIMAT0000730 | MMP9 | 4318 | Western Blot | tarbase |
| hsa-mir-494-3p | MIMAT0002816 | MMP9 | 4318 | Western Blot | tarbase |
| hsa-mir-497-5p | MIMAT0002820 | MMP9 | 4318 | Western Blot | tarbase |
| hsa-mir-655-3p | MIMAT0003331 | MMP9 | 4318 | Western Blot, ELISA | tarbase |
| hsa-mir-1-3p | MIMAT0000416 | MMP9 | 4318 | RPF-Seq, RNA-Seq | tarbase |
| hsa-mir-101-3p | MIMAT0000099 | MMP9 | 4318 | Microarrays | tarbase |
| hsa-mir-146a-5p | MIMAT0000449 | MMP9 | 4318 | Microarrays | tarbase |
| hsa-mir-214-3p | MIMAT0000271 | MMP9 | 4318 | Microarrays | tarbase |
| hsa-mir-330-3p | MIMAT0000751 | MMP9 | 4318 | Microarrays | tarbase |
| hsa-mir-7-5p | MIMAT0000252 | MMP9 | 4318 | Microarrays | tarbase |
| hsa-mir-99b-5p | MIMAT0000689 | MMP9 | 4318 | Microarrays | tarbase |
| hsa-mir-374a-5p | MIMAT0000727 | MMP9 | 4318 | Microarrays | tarbase |
| hsa-mir-122-5p | MIMAT0000421 | BCL6 | 604 | Biotin-Microarrays | tarbase |
| hsa-mir-126-5p | MIMAT0000444 | BCL6 | 604 | HITS-CLIP | tarbase |
| hsa-mir-149-5p | MIMAT0000450 | BCL6 | 604 | HITS-CLIP | tarbase |
| hsa-mir-193a-3p | MIMAT0000459 | BCL6 | 604 | HITS-CLIP | tarbase |
| hsa-mir-22-5p | MIMAT0004495 | BCL6 | 604 | HITS-CLIP | tarbase |
| hsa-mir-25-3p | MIMAT0000081 | BCL6 | 604 | HITS-CLIP | tarbase |
| hsa-mir-26a-5p | MIMAT0000082 | BCL6 | 604 | Western Blot | tarbase |
| hsa-mir-30a-5p | MIMAT0000087 | BCL6 | 604 | HITS-CLIP | tarbase |
| hsa-mir-30b-3p | MIMAT0004589 | BCL6 | 604 | Luciferase Reporter Assay | tarbase |
| hsa-mir-30c-5p | MIMAT0000244 | BCL6 | 604 | HITS-CLIP | tarbase |
| hsa-mir-30d-5p | MIMAT0000245 | BCL6 | 604 | HITS-CLIP | tarbase |
| hsa-mir-30e-5p | MIMAT0000692 | BCL6 | 604 | HITS-CLIP | tarbase |
| hsa-mir-379-5p | MIMAT0000733 | BCL6 | 604 | HITS-CLIP | tarbase |
| hsa-mir-409-5p | MIMAT0001638 | BCL6 | 604 | HITS-CLIP | tarbase |
| hsa-mir-625-5p | MIMAT0003294 | BCL6 | 604 | HITS-CLIP | tarbase |
| hsa-mir-628-5p | MIMAT0004809 | BCL6 | 604 | HITS-CLIP | tarbase |
| hsa-mir-876-5p | MIMAT0004924 | BCL6 | 604 | HITS-CLIP | tarbase |
| hsa-mir-92a-1-5p | MIMAT0004507 | BCL6 | 604 | HITS-CLIP | tarbase |
| hsa-mir-92a-3p | MIMAT0000092 | BCL6 | 604 | HITS-CLIP | tarbase |
| hsa-mir-93-5p | MIMAT0000093 | BCL6 | 604 | PAR-CLIP | tarbase |
| hsa-mir-27a-3p | MIMAT0000084 | BCL6 | 604 | HITS-CLIP, Microarrays | tarbase |
| hsa-mir-27b-3p | MIMAT0000419 | BCL6 | 604 | HITS-CLIP | tarbase |
| hsa-mir-4701-3p | MIMAT0019799 | BCL6 | 604 | HITS-CLIP | tarbase |
| hsa-mir-485-5p | MIMAT0002175 | BCL6 | 604 | HITS-CLIP | tarbase |
| hsa-mir-10b-5p | MIMAT0000254 | BCL6 | 604 | Microarrays | tarbase |
| hsa-mir-129-2-3p | MIMAT0004605 | BCL6 | 604 | Microarrays | tarbase |
| hsa-mir-203a-3p | MIMAT0000264 | BCL6 | 604 | Microarrays | tarbase |
| hsa-mir-34b-5p | MIMAT0000685 | BCL6 | 604 | Microarrays | tarbase |
| hsa-mir-424-5p | MIMAT0001341 | BCL6 | 604 | Luciferase Reporter Assay | tarbase |
| hsa-mir-503-5p | MIMAT0002874 | BCL6 | 604 | Luciferase Reporter Assay | tarbase |
| hsa-let-7a-5p | MIMAT0000062 | ETS2 | 2114 | HITS-CLIP | tarbase |
| hsa-mir-103a-3p | MIMAT0000101 | ETS2 | 2114 | HITS-CLIP | tarbase |
| hsa-mir-107 | MIMAT0000104 | ETS2 | 2114 | HITS-CLIP | tarbase |
| hsa-mir-10a-3p | MIMAT0004555 | ETS2 | 2114 | HITS-CLIP | tarbase |
| hsa-mir-1180-3p | MIMAT0005825 | ETS2 | 2114 | HITS-CLIP | tarbase |
| hsa-mir-126-3p | MIMAT0000445 | ETS2 | 2114 | HITS-CLIP | tarbase |
| hsa-mir-146a-3p | MIMAT0004608 | ETS2 | 2114 | HITS-CLIP | tarbase |
| hsa-mir-17-5p | MIMAT0000070 | ETS2 | 2114 | HITS-CLIP | tarbase |
| hsa-mir-1915-5p | MIMAT0007891 | ETS2 | 2114 | HITS-CLIP | tarbase |
| hsa-mir-200c-5p | MIMAT0004657 | ETS2 | 2114 | HITS-CLIP | tarbase |
| hsa-mir-216a-5p | MIMAT0000273 | ETS2 | 2114 | HITS-CLIP | tarbase |
| hsa-mir-221-3p | MIMAT0000278 | ETS2 | 2114 | Luciferase Reporter Assay, qPCR | tarbase |
| hsa-mir-222-3p | MIMAT0000279 | ETS2 | 2114 | Western Blot | tarbase |
| hsa-mir-22-5p | MIMAT0004495 | ETS2 | 2114 | HITS-CLIP | tarbase |
| hsa-mir-2467-5p | MIMAT0019952 | ETS2 | 2114 | HITS-CLIP | tarbase |
| hsa-mir-26a-5p | MIMAT0000082 | ETS2 | 2114 | HITS-CLIP | tarbase |
| hsa-mir-298 | MIMAT0004901 | ETS2 | 2114 | HITS-CLIP | tarbase |
| hsa-mir-34a-5p | MIMAT0000255 | ETS2 | 2114 | PAR-CLIP | tarbase |
| hsa-mir-374a-5p | MIMAT0000727 | ETS2 | 2114 | PAR-CLIP | tarbase |
| hsa-mir-4254 | MIMAT0016884 | ETS2 | 2114 | HITS-CLIP | tarbase |
| hsa-mir-4286 | MIMAT0016916 | ETS2 | 2114 | HITS-CLIP | tarbase |
| hsa-mir-4448 | MIMAT0018967 | ETS2 | 2114 | PAR-CLIP | tarbase |
| hsa-mir-522-5p | MIMAT0005451 | ETS2 | 2114 | IMPACT-Seq | tarbase |
| hsa-mir-628-5p | MIMAT0004809 | ETS2 | 2114 | HITS-CLIP | tarbase |
| hsa-let-7b-5p | MIMAT0000063 | ETS2 | 2114 | HITS-CLIP | tarbase |
| hsa-let-7c-5p | MIMAT0000064 | ETS2 | 2114 | HITS-CLIP | tarbase |
| hsa-let-7e-5p | MIMAT0000066 | ETS2 | 2114 | HITS-CLIP | tarbase |
| hsa-let-7f-5p | MIMAT0000067 | ETS2 | 2114 | HITS-CLIP | tarbase |
| hsa-let-7g-5p | MIMAT0000414 | ETS2 | 2114 | HITS-CLIP | tarbase |
| hsa-let-7i-5p | MIMAT0000415 | ETS2 | 2114 | HITS-CLIP | tarbase |
| hsa-mir-369-3p | MIMAT0000721 | ETS2 | 2114 | HITS-CLIP | tarbase |
| hsa-mir-495-3p | MIMAT0002817 | ETS2 | 2114 | HITS-CLIP | tarbase |
| hsa-mir-98-5p | MIMAT0000096 | ETS2 | 2114 | HITS-CLIP | tarbase |
| hsa-mir-124-3p | MIMAT0000422 | ETS2 | 2114 | Microarrays | tarbase |
| hsa-mir-191-5p | MIMAT0000440 | ETS2 | 2114 | Microarrays | tarbase |
| hsa-mir-210-3p | MIMAT0000267 | ETS2 | 2114 | Microarrays | tarbase |
| hsa-mir-374b-5p | MIMAT0004955 | ETS2 | 2114 | Chimeric fragments | tarbase |
| hsa-mir-204 | MIMAT0000265 | MMP9 | 4318 | other | 20369013 |

Supplementary table 3: 148 miRNAs of 3 diagnostic genes
